# Genomic Amplification of *TBC1D31* Promotes Hepatocellular Carcinoma Through Reducing the Rab22A‐Mediated Endolysosomal Trafficking and Degradation of EGFR

**DOI:** 10.1002/advs.202405459

**Published:** 2024-08-29

**Authors:** Pengbo Cao, Hongxia Chen, Ying Zhang, Qi Zhang, Mengting Shi, Huihui Han, Xiaowen Wang, Liang Jin, Bingqian Guo, Rongjiao Hao, Xi Zhao, Yuanfeng Li, Chengming Gao, Xinyi Liu, Yahui Wang, Aiqing Yang, Chenning Yang, Anfeng Si, Hua Li, Qingfeng Song, Fuchu He, Gangqiao Zhou

**Affiliations:** ^1^ State Key Laboratory of Medical Proteomics National Center for Protein Sciences at Beijing Beijing Institute of Radiation Medicine Beijing 100850 China; ^2^ School of Life Sciences Tsinghua University Beijing 100084 China; ^3^ University of South China Hengyang 421001 China; ^4^ Guangxi Medical University Nanning 530021 China; ^5^ State Key Laboratory of Medical Proteomics National Center for Protein Sciences at Beijing Beijing Institute of Lifeomics Beijing 102206 China; ^6^ Hebei University Baoding 071000 China; ^7^ Jinling Hospital Affiliated Hospital of Medical School Nanjing University Nanjing 210002 China; ^8^ Department of Oncology Chengdu Military General Hospital Chengdu 610083 China; ^9^ Affiliated Cancer Hospital of Guangxi Medical University Nanning 530021 China

**Keywords:** 8q24.13 amplification, epidermal growth factor receptor (EGFR) trafficking, hepatocellular carcinomas (HCCs), lenvatinib, Rab22A, TBC1D31

## Abstract

Hepatocellular carcinomas (HCCs) are characterized by a vast spectrum of somatic copy number alterations (CNAs); however, their functional relevance is largely unknown. By performing a genome‐wide survey on prognosis‐associated focal CNAs in 814 HCC patients by an integrative computational framework based on transcriptomic data, genomic amplification is identified at 8q24.13 as a promising candidate. Further evidence is provided that the 8q24.13 amplification‐driven overexpression of Rab GTPase activating protein TBC1D31 exacerbates HCC growth and metastasis both in vitro and in vivo through activating Epidermal growth factor receptor (EGFR) signaling. Mechanistically, TBC1D31 acts as a Rab GTPase activating protein to catalyze GTP hydrolysis for Rab22A and then reduces the Rab22A‐mediated endolysosomal trafficking and degradation of EGFR. Notably, overexpression of TBC1D31 markedly increases the resistance of HCC cells to lenvatinib, whereas inhibition of the TBC1D31‐EGFR axis can reverse this resistance phenotype. This study highlights that *TBC1D31* at 8q24.13 is a new critical oncogene, uncovers a novel mechanism of EGFR activation in HCC, and proposes the potential strategies for treating HCC patients with *TBC1D31* amplification or overexpression.

## Graphical abstract

Epidermal growth factor receptor (EGFR), rarely driven by oncogenic mutations (<5%) in hepatocellular carcinoma (HCC), soars in protein levels (> 60%). Here, TBC1D31 is identified as a novel Rab GTPase activating protein to catalyze GTP hydrolysis for Rab22A, which mediates endolysosomal trafficking and degradation of EGFR, and proposes TBC1D31 as a novel actionable target for HCC patients with 8q24.13 amplification.

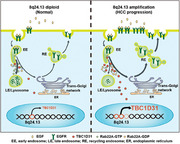

## 1 Introduction

Hepatocellular carcinoma (HCC), which accounts for more than 90% of liver cancers, is the third leading cause of cancer‐related mortality worldwide.^[^ ^1^ ^]^ Especially, China accounts for over 50% of all newly diagnosed HCC cases and deaths.^[^ ^2^ ^]^ Despite in‐depth and extensive research on new prevention and therapeutic strategies for HCC, the 5‐year survival rate of HCC patients remains to be limited.^[^ ^3^ ^]^ Thus, there is an urgent need to further explore novel functional targets for the treatment of HCC.

Due to high genomic heterogeneity, recent large‐scale DNA sequencing studies have detected diverse but low‐frequency oncogenic mutations in numerous genes in HCC.^[^ ^4^ ^]^ Alternatively, extensive genomic analyses have revealed many copy number alterations (CNAs) in the HCC genomes, such as 11q13.3 (*FGF19*), 7q31.2 (*MET*), and 6p21.1 (*VEGFA*) amplifications, leading to substantial advances in identifying cancer drivers.^[^ ^5^, ^6^, ^7^, ^8^ ^]^ However, most CNAs, especially those that span large genomic regions containing multiple candidate genes, have no functional clues to pathogenesis of diseases and/or have largely not been explored. Additionally, numerous studies are restricted to a limited sample size and cannot illustrate the landscape of reproducible HCC‐associated CNAs, therefore posing a challenge in translating them into medical practice. CNAs can affect gene expression at the same locus via the cis‐regulation effects in a dosage‐dependent manner. Thus, several computational methods have been developed to investigate the genomic CNAs in cancers by inferring CNAs using transcriptional data, such as ACE (analysis of CNAs by expression data) and WACE (wavelet based algorithm to analyze CNAs based on expression).^[^ ^9^, ^10^ ^]^ These algorithms consider the subsequent effects of CNAs on gene expression patterns, therefore enabling the identification of candidate critical genes that are heavily driven by CNAs, especially for those large‐size CNAs. Notably, previous extensive studies have generated a large amount of transcriptional data for HCC, therefore providing an opportunity to explore the promising CNAs in a large sample of this malignancy.

Epidermal growth factor receptor (EGFR) is a member of the receptor tyrosine kinases (RTKs) family and plays critical roles in the malignant transformation and metastasis of cancers.^[^ ^11^ ^]^ Numerous genomic studies have shown that *EGFR* is recurrently disrupted by genomic alterations in various types of cancer.^[^ ^12^ ^]^ With regard to HCC, less than 5% of patients have oncogenic mutations or amplifications in *EGFR*.^[^ ^4^ ^]^ However, EGFR protein was found to be commonly overexpressed in > 60% of human HCCs and significantly correlated with the aggressiveness of patients.^[^ ^13^ ^]^ This astonishing inconsistency suggests that there may exist alternative mechanism(s) accounting for the overexpression of EGFR protein in HCC. Emerging evidence has shown that the defective endocytic trafficking of EGFR by a group of Rab GTPases has been recognized as a novel mechanism for regulating the expression and activity of EGFR.^[^ ^14^, ^15^, ^16^, ^17^ ^]^ However, multiple facets of the EGFR trafficking mechanism in HCC cells are still far from being elucidated.

Here, we analyzed a large collection of transcriptomic datasets which consist of 814 HCC patients. By using the ACE method, we identified a total of 15 significant somatic HCC‐associated CNAs. Among them, the chromosomal 8q24.13 amplification was shown to be significantly correlated with poor clinical outcomes of patients. Through high‐content functional screening and subsequent series of functional assays, *TBC1D31* within 8q24.13 amplification was shown to function as a novel oncogene in the development of HCC. Mechanistically, TBC1D31 delays EGFR degradation through hydrolyzing Rab22A, which has been shown to act as a promoter in the trafficking of EGFR from early endosomes to late endosome/lysosomes for degradation, thereby enhancing the EGFR signaling in HCC cells. Moreover, we found that either downregulating TBC1D31 or inhibiting EGFR can sensitize HCC cells to lenvatinib, therefore providing potential therapeutic strategies for HCC.

## 2 Results

### 2.1 Genomic Amplification at 8q24.13 Confers Poor Clinical Outcomes in HCC Patients

We collected a large collection of transcriptomic datasets of 814 pairs of tumor tissues and adjacent non‐tumor liver tissues from six independent cohorts of HCC patients (discovery cohorts 1 – 6 [DISC1 – 6]; Table S1, Supporting Information). Then, the analysis of CNAs by expression data (ACE) algorithm,^[^ ^9^ ^]^ which uses the gene expression change‐based geometry‐weighted neighborhood scores (NSs) for inferring the regional CNAs, was used to identify the somatic CNAs (Figure S1a, Supporting Information). Finally, a total of 15 significant common focal CNAs were identified, including eight amplifications and seven deletions (Table S2, Supporting Information). The genomic intervals spanned by these 15 focal CNAs ranged from 0.7 to 15.9 mega base (Mb), and a total of 427 genes were affected by these CNAs. Among these 15 CNAs, 13 ones have been reported by previous studies.^[^ ^5^, ^7^, ^8^ ^]^ The other two ones, i.e., the amplification at 20q11.21‐q11.22 and the deletion at 12p13.31‐p13.2, were identified for the first time here in HCC.

**Figure 1:**
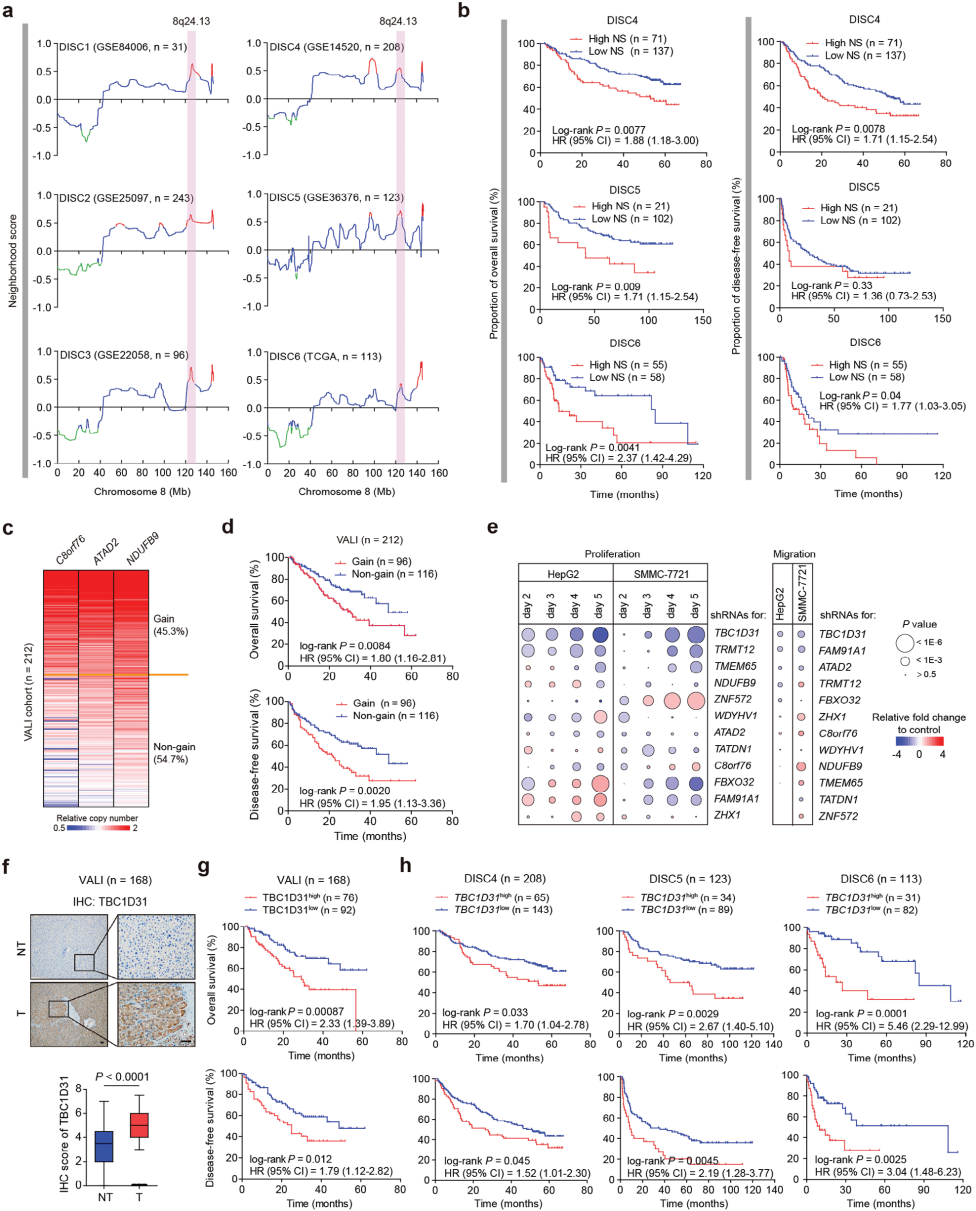
Genomic amplification at chromosome 8q24.13 predicts poor clinical outcomes in HCC patients. a) Genomic amplification at 8q24.13 was detected by ACE method in HCCs from the discovery cohorts (DISC1 – 6). The traces are the neighborhood scores (NSs) on chromosome 8 produced by ACE method. Red and green peaks represent the significant genomic amplifications and deletions, respectively. ACE, the analysis of CNAs by expression data. b) Kaplan‐Meier plots of the overall survival (OS) and DFS rates of HCC patients stratified by NSs at 8q24.13 amplification region (high vs low; log‐rank test) from the DISC4, 5, and 6 cohorts. The samples with significant NS at the 8q24.13 locus determined by single‐sample assessment were assigned to the “high” group, while the others were assigned to the “low” group. HR, hazard ratio; CI, confidence interval. c) Genotyping of the 8q24.13 amplification using the real‐time quantitative PCR (qPCR) assays in HCCs from the validation cohort (VALI; n = 212). The average copy number of three genes (*C8orf76*, *ATAD2*, and *NDUFB9*) was used as the readout. A relative copy number > 1.25 was defined as genomic gain. d) Kaplan‐Meier plots of the OS and DFS rates of HCC patients stratified by 8q24.13 copy numbers (gain vs non‐gain) from the VALI cohort. e) Summary plot of the high‐content functional screening assays for assessing those 12 genes located at 8q24.13 amplification region. The abilities of cell proliferation and migration were determined by cell number counting assays and wound healing assays, respectively, in HepG2 and SMMC‐7721 cells. f) Immunohistochemistry (IHC) analyses of TBC1D31 in HCC tissues (T) and non‐tumor liver tissues (NT) from the VALI cohort (n = 168). Scale bars, 100 µm. The *P* value was assessed by Wilcox rank sum test. g) Kaplan‐Meier plots of the OS and DFS rates of HCC patients from the VALI cohort stratified by TBC1D31 protein levels (high vs low). Samples with the IHC score of TBC1D31 ≥ 5 were defined as the TBC1D31^high^ group, while the others as the TBC1D31^low^ group. h) Kaplan‐Meier plots of the OS and DFS rates of HCC patients stratified by *TBC1D31* mRNA levels (high vs low) from the three discovery cohorts (DISC4, 5, and 6). Samples with the relative fold change (tumor/non‐tumor) of *TBC1D31* mRNA expression levels ≥ 2 were defined as the *TBC1D31* ^high^ group while the others as the *TBC1D31* ^low^ group.

Next, we assessed the clinical relevance in three discovery cohorts (including DISC4 – 6) with follow‐up information available. Only the amplification at 8q24.13 consistently predicts a significantly shorter over survival (OS) rate in all three cohorts (*P* = 0.0077 in DISC4, 0.0090 in DISC5, and 0.0041 in DISC6, respectively; **Figure** **1**a,b; Table S3, Supporting Information). It also predicts shorter disease‐free survival (DFS) rate in the DISC4 and DISC6 cohorts (*P* = 0.0078 in DISC4 and 0.040 in DISC6, respectively; Figure 1b). Notably, this transcriptome‐inferred NS was highly concordant with the relative copy number profiled by genomic arrays (e.g., single nucleotide polymorphism [SNP] genotyping arrays or comparative genomic hybridization [CGH] arrays) in the DISC1, DISC4, and DISC6 cohorts (all *r* ≥ 0.8 and *P* < 0.001; Figure S1b,c, Supporting Information). Thus, these findings suggest that the amplification at 8q24.13 is a strong indicator of poor prognosis in HCC patients, and merits further investigation.

We further evaluated the clinical relevance in another independent HCC cohort (validation [VALI] cohort; n = 212; Table S4, Supporting Information) by using genomic DNA qPCR assays. Three pairs of primers were designed to amplify DNA sequences at both ends and the middle of the 8q24.13 amplified interval (e.g., *C8orf76*, *ATAD2* and *NDUFB9*). Ninety‐six out of 212 HCCs (45.3%) harbor the 8q24.13 genomic gain (Figure 1c; Table S4, Supporting Information), mainly male patients (*P* = 0.011; Table S5, Supporting Information). Kaplan‐Meier analyses showed that the HCC patients with 8q24.13 gain have reduced OS and DFS rates (*P* = 0.0084 and *P* = 0.0020, respectively; Figure 1d) compared to those without 8q24.13 gain. Multivariate Cox proportional hazards regression analyses further revealed its independent prognostic value in HCC patients (*P* = 0.045 and *P* = 0.016, respectively; Table S6, Supporting Information). Intriguingly, pan‐cancer analyses on the basis of the TCGA genomic datasets showed that 8q24.13 is also frequently amplified in many other types of cancer, with the frequency varying from 4% to 74% (Figure S1d, Supporting Information). Again, 8q24.13 genomic gain was significantly associated with reduced OS and/or DFS rates in multiple types of cancer (Figure S1e–n, Supporting Information). Collectively, these results suggest that the genomic amplification at 8q24.13 predicts poor clinical outcomes in patients with HCC and several other types of cancer.

### 2.2 *TBC1D31* Is a Potential Functional Target Gene within the 8q24.13 Amplification

The minimal common interval of 8q24.13 amplification spans ≈1.91 Mb (from 124154100 to 126060811 base pairs [bp], based on National Center for Biotechnology Information [NCBI] Build 36; Figure 1a), where exist a total of 16 protein‐coding genes (Figure S1b, Supporting Information). Integrated analysis of genomic and transcriptional data (from DISC1, 4, and 6) revealed its strong cis‐effects on expression levels of these 16 genes (Figure S2a,b, Supporting Information). Among them, 12 ones are concordantly elevated in HCC tissues compared to the non‐tumor liver tissues in at least half of the discovery cohorts (Figure S2a, Supporting Information), suggesting their potential relevance in HCC.

We then assessed the tumorigenic effects of these 12 genes in HepG2 and SMMC‐7721 cells using the high‐content screening (HCS) platform. Cell counting assays showed that siRNA‐mediated knockdown of three genes (*TBC1D31*, *TMEM65*, and *TRMT12*) significantly reduces the proliferation of both cell lines (Figure S2c, Supporting Information). The wound healing assays also revealed three candidates (*TBC1D31*, *ATAD2*, and *FAM91A1*) involved in cell migration (Figure S2c, Supporting Information). Among these five candidates, *ATAD2* encodes an ATPase family AAA domain‐containing protein and is involved in the development of multiple types of cancer, including HCC.^[^ ^18^ ^]^ Although the other four genes have not been reported previously to be directly relevant to HCC, they are biologically plausible in tumorigenesis. *TRMT12* is a homolog of a yeast gene encoding a tRNA methyltransferase and has been shown to be a prognosis predictor for patients with head and neck squamous cell carcinoma.^[^ ^19^ ^]^ *FAM91A1*, which encodes a component of the WDR11 complex, acts together with TBC1D23 to facilitate the Golgi‐mediated capture of vesicles.^[^ ^20^ ^]^ *TMEM65*, which encodes a mitochondrial inner‐membrane protein, was involved in the regulation of mitochondrial respiration and colorectal cancer development.^[^ ^21^ ^]^ *TBC1D31* encodes a member of the GTPase‐activating protein family, which was well‐known in vesicle‐mediated transport.^[^ ^22^ ^]^ Notably, we observed that the knockdown of *TBC1D31* has the strongest inhibitory effects on both the proliferation and migration of HCC cells (Figure 1e). Thus, these results made the *TBC1D31* a promising candidate functional target at 8q24.13 amplification locus.

### 2.3 High Expression Levels of *TBC1D31* Predict Poor Clinical Outcomes in HCC Patients

We next assessed whether the TBC1D31 expression levels are relevant to HCC. Compared with the normal human liver cell line L‐02, HCC cell lines show markedly increased levels of TBC1D31, especially those with 8q24.13 gain (e.g., Huh7 and HCCLM3 cells; *r* = 0.83, *P* = 0.039; Figure S2d, Supporting Information). The immunohistochemistry (IHC) analyses showed overexpressed protein levels of TBC1D31 in HCC tissues compared with non‐tumor liver tissues from the VALI cohort (n = 168), which also correlate with the 8q24.13 copy numbers (Figure 1f; Figure S2e, Supporting Information). Additionally, three datasets from the mouse HCC models (including the diethylnitrosamine [DEN]‐induced, CCl_4_‐induced and HBV transgenic models) consistently showed a significant increase in the expression of *Tbc1d31* in liver tumors compared to non‐tumor livers (Figure S2f, Supporting Information). Further, we found that the male or tumor capsule‐free patients, or those with advanced TNM stage, respectively, exhibit significantly higher TBC1D31 expression levels compared to the respective other patients in VALI cohort (Figure S2g and Table S5, Supporting Information). Intriguingly, we observed significantly higher *TBC1D31* expression in liver portal vein tumor thrombus (PVTT) tissues than in their counterpart primary HCC tissues (Figure S2h, Supporting Information). Furthermore, higher TBC1D31 levels in HCC tissues of VALI cohort are significantly correlated with the decreases in OS and DFS rates (Figure 1g). Multivariable Cox proportional hazards regression analyses further revealed the independent prognostic value of TBC1D31 expression in HCC patients (Table S7, Supporting Information). The similar results were also observed in the three discovery cohorts (DISC4 – 6; Figure 1h).

The significant increases in *TBC1D31* mRNA expression in tumor tissues relative to the match non‐tumor tissues were also observed in multiple types of cancer from the TCGA pan‐cancer cohorts (Figure S3a, Supporting Information), and their fold‐changes were significantly correlated with the frequencies of 8q24.13 gain (Figure S3b, Supporting Information). A similar correlation was observed in the CCLE dataset (Figure S3c, Supporting Information). Accordingly, higher levels of TBC1D31 protein were observed in multiple types of cancer indexed in the HPA database (Figure S3d, Supporting Information). Again, TCGA pan‐cancer analyses showed that higher *TBC1D31* mRNA levels are correlated with the advanced clinical stages (Figure S3e, Supporting Information), and poor OS or DFS rates in patients with several other types of cancer (Figure S3f, Supporting Information). Taken together, these findings support the 8q24.13 gain‐driven upregulation of *TBC1D31* as a common event in tumor progression with prognostic value.

### 2.4 TBC1D31 Plays an Oncogenic Role in the Development of HCC

**Figure 2:**
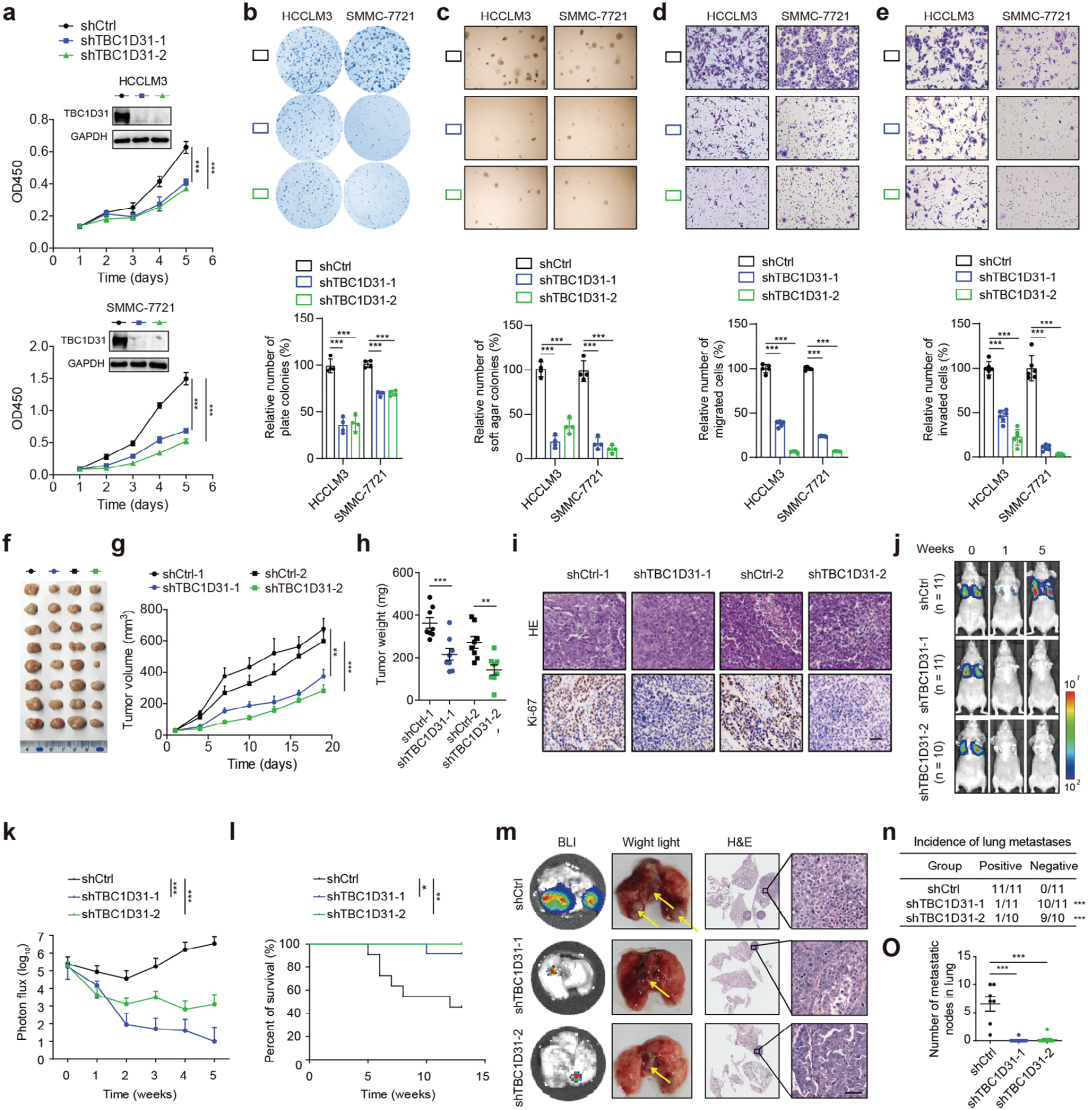
TBC1D31 within the 8q24.13 promotes the tumorigenesis and metastasis of HCC. a–c) The effects of *TBC1D31* knockdown on cell growth, which were determined by Cell Counting Kit‐8 (CCK‐8) (a), plate colony formation (b), and soft agar colony formation (c) assays, respectively, in HCCLM3 and SMMC‐7721 cells. d,e) The effects of *TBC1D31* knockdown on cell migration (d) and invasion (e) in HCCLM3 and SMMC‐7721 cells. Cells that migrated or invaded from the upper well into the lower well of the transwell chamber were stained and counted. f–i) Representative images and volume measurement of control or *TBC1D31*‐knocked‐down HCCLM3 cells‐derived subcutaneous tumors in nude mice (n = 8). The representative images of subcutaneous tumors (f), dynamic change of tumor volume (g), tumor weights (h), and the hematoxylin and eosin (H&E) staining and immunohistochemistry (IHC) staining of Ki‐67 and CD31 proteins in subcutaneous tumors (i) were shown, respectively. Scale bars, 200 µm. j–o) Representative images and metastasis measurement of the nude mice injected with control or *TBC1D31*‐knocked‐down HCCLM3 cells (also expressing luciferase) via the tail veins (n = 10 ∼ 11). j) Representative bioluminescence (BLI) images of mice. k) The dynamic change of photon flux intensity of tumors. l) Kaplan‐Meier plot of survival rates of mice (by log‐rank test). m) Representative BLI images (Left), white‐light images (Middle) and H&E staining (Right) of the metastases in lungs. The yellow arrows on the middle panel indicate the metastatic nodules in lungs. Scale bars, 200 µm. n) The incidence of metastases determined by white‐light images of lungs based on (m). o) The number of metastatic nodules determined by H&E staining in lungs based on (m). Values are expressed as mean ± standard deviation (s.d.) of three or more independent replicates. *P* values were calculated using Student's *t* test unless specifically specified. ^*^, *P* < 0.05; ^**^, *P* < 0.01; ^***^, *P* < 0.001; n.s., not significant.

We then assessed the tumorigenic role of TBC1D31in HCC cells. The shRNA‐mediated knockdown of *TBC1D31* led to a significant reduction in the abilities of cell proliferation, plate clone formation, soft agar colony formation, migration and invasion in SMMC‐7721 and HCCLM3 cells (**Figure** **2**a–e). Conversely, overexpression of TBC1D31 markedly enhanced these abilities in HepG2 and Bel‐7402 cells (Figure S4a–d, Supporting Information).

We further examined the effects of TBC1D31 in vivo. Knockdown of *TBC1D31* in HCCLM3 cells led to a significant decrease in the subcutaneous tumor volume and weight in mice (Figure 2f–h; Figure S4e, Supporting Information). Conversely, TBC1D31 overexpression in HepG2 cells significantly promoted tumor growth (Figure S4f–h, Supporting Information), with the pro‐proliferative effect indicated by increased expression of Ki‐67 (Figure 2i; Figure S4i, Supporting Information). We also used the HCCLM3 cells that express a luciferase reporter to investigate the effects of TBC1D31 on in vivo metastasis. Knockdown of *TBC1D31* in HCCLM3 cells significantly reduced the lung metastasis burden (Figure 2j,k), which consequently resulted in a prolonged survival period of the tumor‐bearing mice (Figure 2l). Histological analyses of the lung metastases also confirmed the inhibitory effect of *TBC1D31* depletion on HCC metastasis (Figure 2m–o). Collectively, these in vitro and in vivo findings indicate that TBC1D31 plays an oncogenic role in the development of HCC.

### 2.5 TBC1D31 Plays Oncogenic Roles through Activating the EGFR Pathway

**Figure 3:**
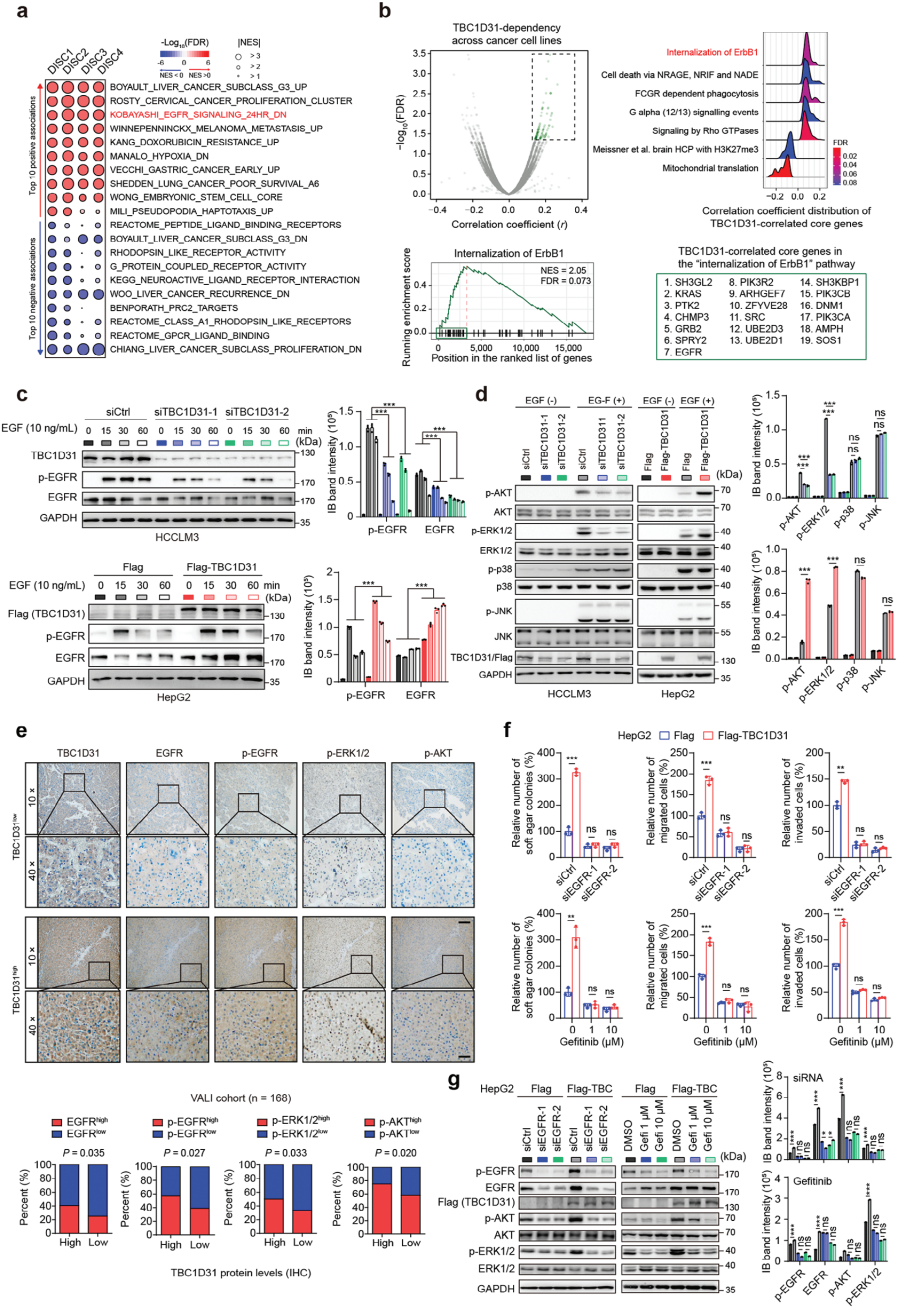
TBC1D31 promotes the activity of the EGFR pathway. a) Gene set enrichment analyses (GSEA) revealed the significant enrichment of gene sets relevant to TBC1D31 based on transcriptomic datasets from the DISC1, 2, 3, and 4 cohorts. GSEA was performed based on the median of *TBC1D31* expression levels (high vs low). The size of the circle represents the normalized enrichment score (NES), and the color represents the scale of ‐log (FDR) with positive NES (red) or negative NES (blue). FDR, false discovery rate. b) Genome‐wide analysis reveals that the genes with dependencies correlated with TBC1D31 are enriched in EGFR pathway. Top left: a total of 17309 gene dependencies were ranked by their correlations with TBC1D31 dependence across 600 cancer lines, as measured by Project Achilles. Spearman coefficient (*r*) versus ‐log_10_(FDR) is plotted for each gene (gray), and the significant direct correlates are highlighted (green, FDR < 5%). Top right: The pathways enriched by the genes with dependencies correlated with TBC1D31 were determined by GSEA (FDR < 0.1). Bottom, the GSEA plot of “Internalization of ErbB1” pathway and the list of TBC1D31‐associated core genes in this term. c) The effects of *TCB1D31* knockdown in HCCLM3 cells (top) or TCB1D31 overexpression in HepG2 cells (bottom) on the levels of total and phosphorylated EGFR (p‐EGFR, Tyr1068) upon EGF treatment (10 ng mL⁻^1^) at the indicated time. d) The effects of *TCB1D31* knockdown in HCCLM3 cells (left) or TCB1D31 overexpression in HepG2 cells (right) on the levels of ERK1/2/p‐ERK1/2 (Thr202/Tyr204), AKT/p‐AKT (Ser473), p38/p‐p38 (Thr180/Tyr182), JNK/p‐JNK (Thr183/Tyr185) upon EGF treatment (10 ng mL⁻^1^) for 30 min (min). e) The percentage of patients harboring the low or high protein levels of EGFR, p‐EGFR, p‐ERK1/2 or p‐AKT, respectively, which is stratified by TBC1D31 protein levels in tumor microarray (TMA; n = 168) derived from the VALI cohort. The IHC score cutoffs for the high‐ and low‐expression stratification were defined as following: TBC1D31, 5; EGFR, 8; p‐EGFR, 3; p‐ERK1/2, 5; and p‐AKT, 7. *P* values were determined by *χ* ^2^ test. f) The siRNAs‐mediated knockdown of *EGFR* (top) or gefitinib (an EGFR inhibitor)‐induced inhibition of EGFR activity (bottom) abolishes the promoting effects by overexpression of TBC1D31 on capacities of soft agar colony formation, migration and invasion in HepG2 cells. g) Knockdown of *EGFR* or treatment with EGFR inhibitor gefitinib (1 and 10 µM) abolishes the promoting effects of TBC1D31 overexpression on the levels of p‐AKT and p‐ERK1/2. Protein levels were determined in HepG2 cells under EGF treatment (10 ng mL⁻^1^) for 30 min after serum starvation. Values are expressed as mean ± standard deviation (s.d.) of three or more independent replicates. *P* values were calculated using Student's *t* test unless specifically specified. ^**^, *P* < 0.01; ^***^, *P* < 0.001; n.s., not significant.

Next, we sought to elucidate the underlying mechanism of TBC1D31's oncogenic role. Gene set enrichment analyses (GSEA) based on gene expression profiles from the DISC1 – 4 cohorts (DISC1, 2, 3 and 4; Table S8, Supporting Information) revealed multiple cancer‐related signatures enriched in TBC1D31^high^ tumors, among which the EGFR pathway (“Kobayashi et al. EGFR signaling 24 h down”) is the most prominent one shared by all cohorts (**Figure** **3**a; Figure S5a,b, Supporting Information). Consistently, by examining the co‐dependencies of *TBC1D31* in the genome‐wide shRNA screening database of 600 cancer cell lines, we found significant enrichment of “Internalization of ErbB1 (EGFR)” by dependent genes (Figure 3b; Table S9, Supporting Information). Given the pivotal roles of EGFR pathway in carcinogenesis,^[^ ^15^ ^]^ we thus hypothesize that TBC1D31 exerts its oncogenic role through this pathway.

Indeed, we observed that knockdown of *TBC1D31* markedly reduces the protein levels of total EGFR and phosphorylated EGFR (p‐EGFR, Tyr1068) in HCCLM3 and SMMC‐7721 cells upon EGF stimulation (Figure 3c; Figure S5c, Supporting Information). Conversely, overexpression of TBC1D31 exhibited a striking and prolonged increase in protein levels of EGFR and p‐EGFR in HepG2 and Bel‐7402 cells (Figure 3c; Figure S5c, Supporting Information). Meanwhile, among the several crucial downstream cascades of EGFR,^[^ ^23^ ^]^ the AKT and ERK1/2 activities were induced by TBC1D31, with markedly increased levels of p‐AKT (Ser473) and p‐ERK1/2 (Thr202/Tyr204) (Figure 3d; Figure S5d, Supporting Information). Consistently, IHC assays showed that TBC1D31 protein levels are significantly positively correlated with the levels of EGFR, p‐EGFR, p‐ERK1/2 and p‐AKT in subcutaneous tumor tissues from nude mice (Figure S5e, Supporting Information), and the HCC tissues from the VALI cohort (n = 168; Figure 3e).

Next, we examined whether the effects of TBC1D31 on the malignant phenotypes and ERK1/2 and AKT activities in HCC cells depend on EGFR. We observed that the promoting effects of TBC1D31 overexpression on soft agar colony formation, migration and invasion abilities, and the levels of p‐ERK1/2 and p‐AKT are abolished when *EGFR* was knocked down by siRNAs in HepG2 and Bel‐7402 cells (Figure 3f,g; Figure S5f, Supporting Information). This EGFR‐dependent role of TBC1D31 was further confirmed by using EGFR inhibitor (EGFRi) gefitinib (Figure 3f,g; Figure S5g, Supporting Information). Together, these findings suggest that TBC1D31 exerts its oncogenic role via activating the EGFR pathway.

### 2.6 TBC1D31 Reduces the Endolysosomal Trafficking and Degradation of EGFR

**Figure 4:**
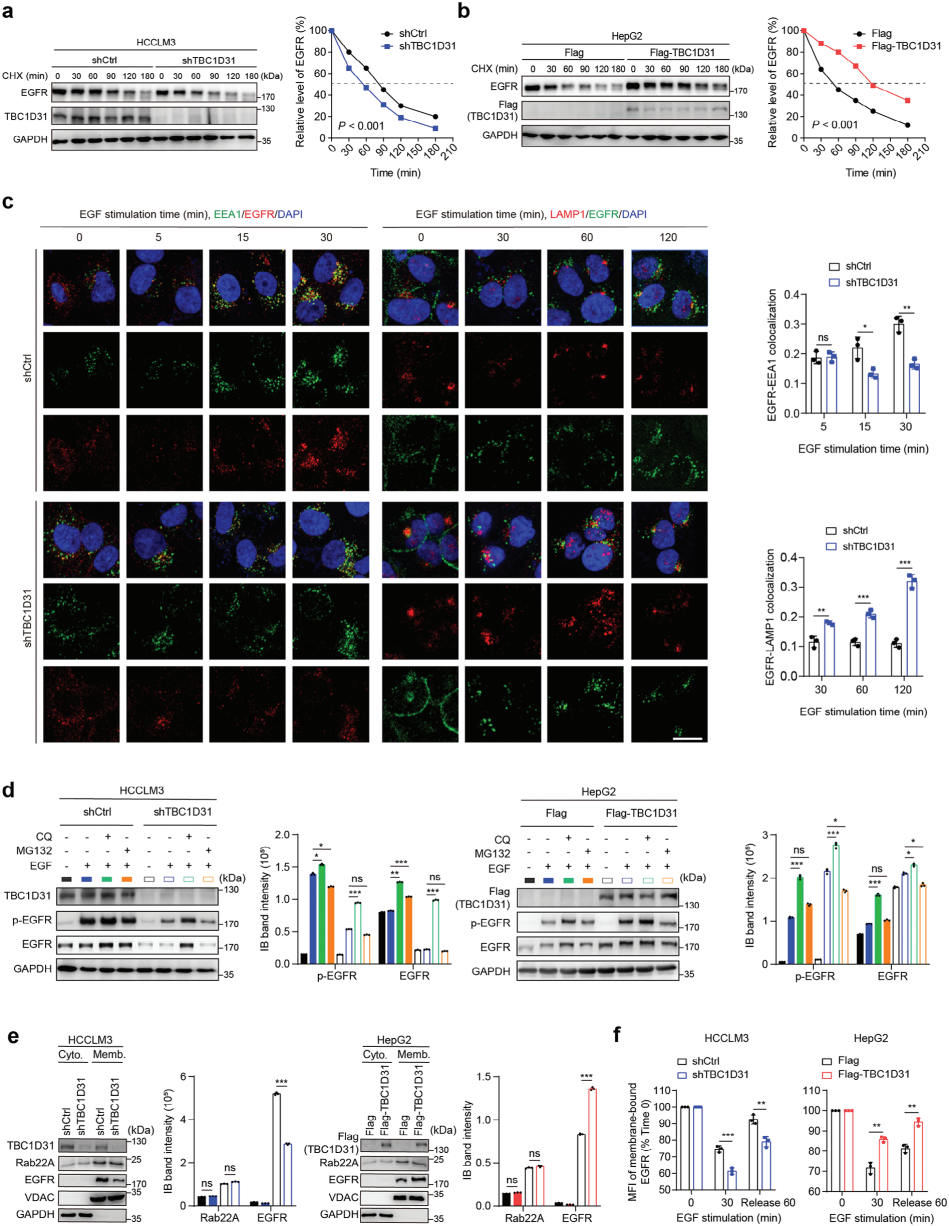
TBC1D31 reduces EGFR degradation by reducing its endolysosomal trafficking. a,b) The half‐life of EGFR protein is shortened by knockdown of *TBC1D31* (shTBC1D31, equally pooled shTBC1D31‐1 and shTBC1D31‐2) in HCCLM3 cells (a) and prolonged by overexpression of TBC1D31 in HepG2 cells (b). CHX, cycloheximide (100 mg mL⁻^1^). c) The effects of *TBC1D31* knockdown on the co‐localization of EGFR and EEA1 or LAMP1 in HCCLM3 cells. The serum‐starved control or *TBC1D31*‐knocked‐down HCCLM3 cells were treated with EGF (10 ng mL⁻^1^). Cells fixed at the different indicated times were imaged using confocal microscope after co‐staining with antibodies against EGFR, EEA1 (an early endosome marker) or LAMP1 (a lysosome marker). Scale bar, 20 µm. d) The effects of lysosome inhibitor chloroquine (CQ, 40 µM) or proteasome inhibitor MG132 (40 µM) treatment on the promoting role of TBC1D31 in the total and phosphorylated EGFR (p‐EGFR) levels in HCCLM3 (left) and HepG2 cells (right). e) The effects of *TBC1D31* knockdown in HCCLM3 cells (left) or TBC1D31 overexpression in HepG2 cells (right) on the levels of EGFR on the cell membrane measured by cell fractionation assays, respectively. f) The effects of *TBC1D31* knockdown in HCCLM3 cells (left) or TBC1D31 overexpression in HepG2 cells (right) on the levels of membrane‐bound EGFR determined by flow cytometry assays, respectively. The recycling ratio of EGFR bound to the membrane in flow cytometry was calculated using the median fluorescence intensity (MFI). Values are expressed as mean ± standard deviation (s.d.) of three or more independent replicates. *P* values were calculated using Student's *t* test unless specifically specified. ^*^, *P* < 0.05; ^**^, *P* < 0.01; ^***^, *P* < 0.001; n.s., not significant.

Next, we investigated how TBC1D31 induces the EGFR expression. We observed that the *EGFR* mRNA levels remain constant after either overexpression or knockdown of *TBC1D31* (Figure S6a, Supporting Information). However, the cycloheximide (CHX) studies showed that the half‐life period of EGFR protein is shortened in *TBC1D31*‐knocked‐down HCCLM3 cells (**Figure** **4**a). Conversely, overexpression of TBC1D31 in HepG2 cells led to a significantly extended half‐life of EGFR protein (Figure 4b).

TBC1D31 is a member of the protein family containing the Tre2/Bub2/Cdc16 (TBC) domain, which is often expected to act as the Rab GTPase activating proteins (GAPs). Numerous GAPs have been shown to catalyze the transition of specific Rab GTPases from an active GTP‐binding state to an inactive GDP‐binding state, thus being the master regulators for membrane receptors endocytosis, trafficking, and recycling.^[^ ^22^ ^]^ We therefore hypothesize that TBC1D31 may regulate EGFR protein levels through a similar process.

To this end, we first tracked the subcellular localization dynamics of EGFR upon EGF stimulation using immunofluorescence assays. After 60 or 120 min of EGF stimulation, EGFR can be delivered to the perinuclear region of *TBC1D31*‐knocked‐down HCCLM3 cells, but remains dispersed in control cells (Figure S6b, Supporting Information). We further measured the co‐localization of EGFR with endosomal markers. After 5 min of EGF treatment, knockdown of *TBC1D31* does not affect the co‐localization of EGFR with the early endosome marker early endosome antigen 1 (EEA1), excluding the involvement in the initial EGFR endocytosis (Figure 4c). However, knockdown of *TBC1D31* attenuated the co‐localization of EGFR with EEA1 after EGF treatment for 15 or 30 min (Figure 4c). Furthermore, the knockdown of *TBC1D31* induced a significant increase in co‐localization between EGFR and the lysosome marker lysosomal associated membrane protein 1 (LAMP1) after EGF treatment for 30, 60, or 120 min (Figure 4c). This time interval corresponds to the transition of the internalized EGFR from early endosomes to late endosomes and ultimately to lysosomal degradation. We also examined both EEA1 and LAMP1 protein expression levels after knocking down *TBC1D31* and found no significant changes, suggesting that TBC1D31 does not affect EEA1 or LAMP1 expression levels but rather influences the colocalization of EGFR with EEA1 and LAMP1. Further, the downregulation of EGFR protein induced by *TBC1D31* knockdown in HCCLM3 cells can be blocked by the lysosomal inhibitor chloroquine (CQ), but not by the proteasomal inhibitor MG132 (Figure 4d). Consistently, treatment of CQ further enhanced the accumulation of EGFR in TBC1D31‐overexpressed HepG2 cells (Figure 4d). Together, these findings suggest that TBC1D31‐mediated EGFR accumulation is, at least partially, dependent on an endolysosome‐mediated pathway, but not a transcriptional effect or ubiquitin‐proteasome pathway.

Furthermore, by performing cell fractionation assays, we observed that *TBC1D31* knockdown reduces the accumulation of membrane‐bound EGFR in HCCLM3 cells; whereas TBC1D31 overexpression displays the opposite effect in HepG2 cells (Figure 4e). Similar effects were obtained by flow cytometry assays (Figure 4f). Collectively, these results suggest that TBC1D31 reduces the trafficking of EGFR from the early endosomes to the late endosomes/lysosomes for degradation, thereby guiding more EGFR recycled to the cell membrane.

### 2.7 TBC1D31 Interacts with Rab22A and Reduces the Rab22A‐Mediated Endolysosomal Trafficking of EGFR

**Figure 5:**
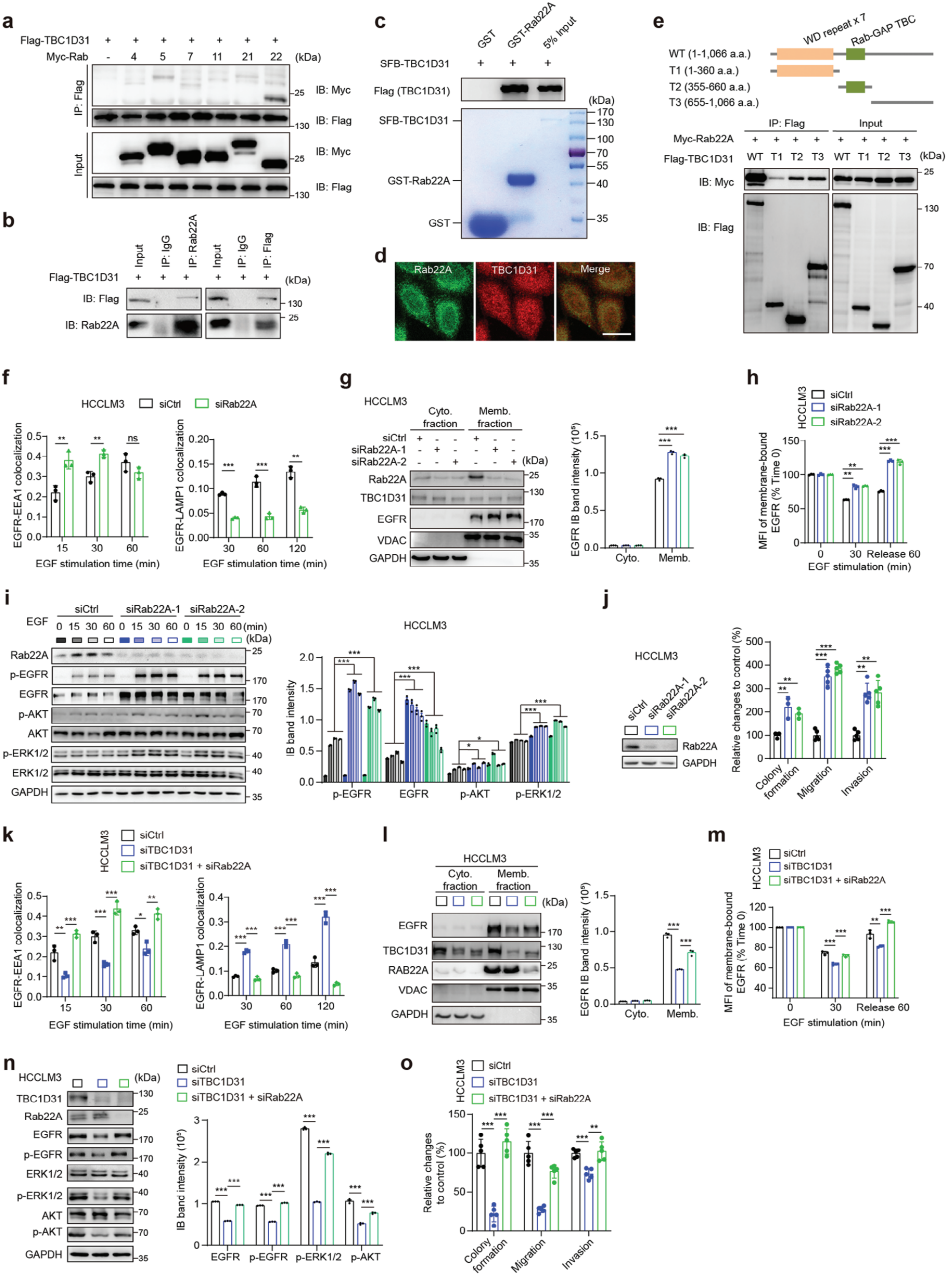
TBC1D31 interacts with Rab22A and reduces the Rab22A‐mediated endolysosomal trafficking of EGFR. a) Co‐immunoprecipitation (Co‐IP) assays detecting the interaction of Flag‐TBC1D31 with Myc‐Rab4, ‐Rab5, ‐Rab7, ‐Rab11, ‐Rab21 and ‐Rab22A in HEK293T cells. b) Co‐IP assays confirming the interaction between Flag‐TBC1D31 and the endogenous Rab22A in HepG2 cells. IP and immunoblotting (IB) assays were performed using the anti‐mouse Flag and anti‐rabbit Rab22A, respectively. The rabbit (left panel) or mouse (right panel) IgG was used as the negative controls. c) The in vitro interaction between the TBC1D31 (GST‐tagged) and Rab22A (SFB‐tagged) is evaluated by GST pull‐down assays. kD, kilodalton. d) Co‐localization of endogenous TBC1D31 and Rab22A proteins at cytoplasm in HepG2 cells by multi‐colored immunofluorescence assays. Scale bar, 20 µm. e) IB assays of Myc‐tagged Rab22A pulled down by serial deletions of TBC1D31. The schematic diagram of full‐length and deleted fragments of TBC1D31 is shown above. a.a., amino acid; IB, immunoblotting; WT, wild type. f) The effects of *Rab22A* knockdown on the co‐localization of EGFR and EEA1 or LAMP1 in HCCLM3 cells. g) The effects of *Rab22A* knockdown in HCCLM3 cells on the levels of EGFR on the cell membrane measured by cell fractionation assays. h) The effects of *Rab22A* knockdown in HCCLM3 cells on the levels of membrane‐bound EGFR determined by flow cytometry assays. i) The effects of *Rab22A* knockdown in HCCLM3 cells on the EGFR accumulation and the activation of downstream cascades upon EGF treatment (10 ng mL⁻^1^) for the indicated time. j) The effects of *Rab22A* knockdown in HCCLM3 cells on the abilities of cells soft agar colony formation, migration and invasion. k) The effect of *Rab22A* knockdown on the role of TBC1D31 inhibition in regulating the co‐localization of EGFR and EEA1 or LAMP1 in HCCLM3 cells. Scale bar, 20 µm. l,m) The effect of *Rab22A* knockdown on the role of TBC1D31 inhibition in reducing the levels of EGFR on the cell membrane, as assessed by cell fractionation assays (l) and flow cytometry assays (m) in HCCLM3 cells. n) The effect of *Rab22A* knockdown on the role of TBC1D31 inhibition in attenuating the EGFR accumulation and the activation of downstream cascades in HCCLM3 cells. Protein levels were determined at 30 min after EGF stimulation (10 ng mL⁻^1^). o) The effect of *Rab22A* knockdown on the role of TBC1D31 inhibition in attenuating the abilities of cell soft agar colony formation, migration, and invasion in HCCLM3 cells. Values are expressed as mean ± standard deviation (s.d.) of three or more independent replicates. *P* values were calculated using Student's *t* test unless specifically specified. ^*^, *P* < 0.05; ^**^, *P* < 0.01; ^***^, *P* < 0.001; n.s., not significant.

Next, we sought to illuminate how TBC1D31 reduces the endolysosomal trafficking of EGFR. Several well‐studied Rab GTPases, including Rab4A, Rab5A, Rab7A, Rab11A, Rab21A, and Rab22A, have been shown to be involved in EGFR trafficking.^[^ ^24^, ^25^, ^26^, ^27^ ^]^ We, therefore, explored the potential bindings of these Rabs with TBC1D31 using co‐immunoprecipitation (co‐IP) assays in HEK293 cells. Indeed, we observed that Flag‐TBC1D31 can bind with Myc‐Rab22A, but not with the other Rabs (**Figure** **5**a). This interaction was replicated by co‐IP assays in HepG2 cells (Figure 5b). Consistently, both glutathione S‐transferase (GST) pull‐down and S protein‐Flag‐Streptavidin binding peptide (SFB) pull‐down assays showed that TBC1D31 directly binds to Rab22A in vitro (Figure 5c; Figure S7a, Supporting Information). Immunofluorescence assays also demonstrated the cytoplasmic co‐localization of TBC1D31 with its binding partner Rab22A in HepG2 cells (Figure 5d). Then, we performed protein domain mapping experiments using the truncated TBC1D31, and found the GAP domain and C terminal are required for the TBC1D31‐Rab22A interaction (Figure 5e).

We then investigated the tumorigenic role of Rab22A in HCC cells. Contrary to the effects of *TBC1D31* inhibition, the knockdown of *Rab22A* significantly enhanced the co‐localization of EGFR and EEA1, while reducing the co‐localization of EGFR and LAMP1 in HCCLM3 cells (Figure 5f). The cell fractionation assays and flow cytometry assays consistently showed that knockdown of *Rab22A* significantly increases the levels of membrane‐bound EGFR in HCCLM3 cells (Figure 5g,h), whereas overexpression of Rab22A has the opposite effects in HepG2 cells (Figure S7b,c, Supporting Information). Accordingly, knockdown of *Rab22A* significantly enhanced the levels of EGFR, p‐EGFR, p‐AKT and p‐ERK1/2, and the malignant phenotypes in HCCLM3 and Bel‐7402 cells (Figure 5i,j; Figure S7d,e, Supporting Information), whereas overexpression of Rab22A has the opposite effects in HepG2 and Bel‐7402 cells (Figure S7f,g, Supporting Information). Together, these findings suggest that Rab22A facilitates the endolysosomal trafficking and recycling of EGFR, and thereby reducing the EGFR pathway activity and malignant phenotypes in HCC cells.

Finally, we observed that the effects of *TBC1D31* knockdown on the EGFR trafficking and activity, and the malignant phenotypes are almost entirely abolished in *Rab22A*‐knocked‐down HCCLM3 cells (Figure 5k–o). Similar results were observed in Bel‐7402 cells (Figure S7h–k, Supporting Information). These findings thus suggest that TBC1D31 exerts its roles dependent on Rab22A.

### 2.8 TBC1D31 Functions Dependent on Its Catalytic Effect on Rab22A

**Figure 6:**
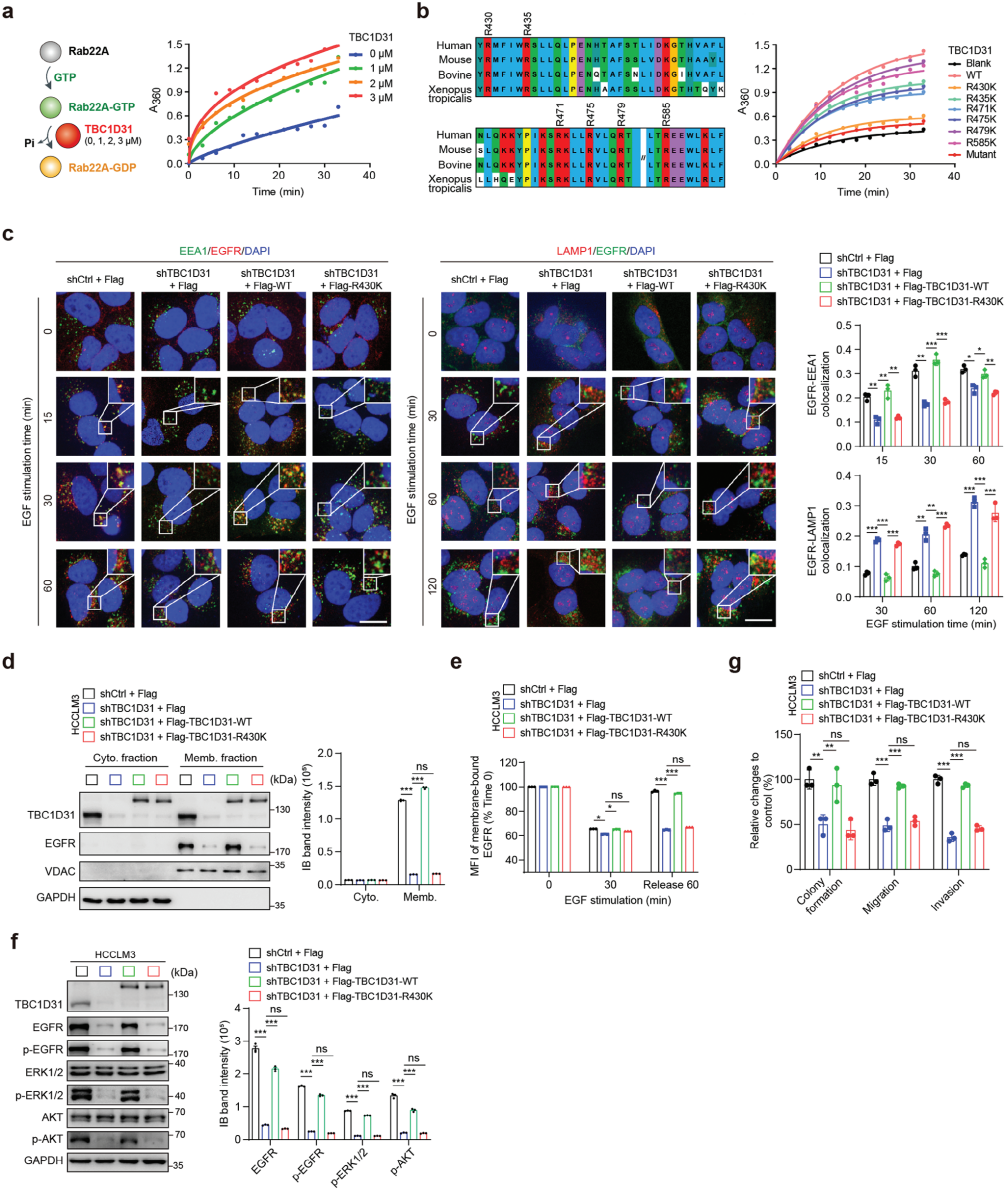
TBC1D31 functions dependent on its catalytic effect on Rab22A. a) TBC1D31 shows the GTPase‐activating protein (GAP) activity against Rab22A in vitro. The single‐turnover kinetics of TBC1D31‐mediated GTP hydrolysis of GTP‐loaded Rab22A (0.2 µM) was measured by the continuous enzyme‐coupled optical assay for the release of inorganic phosphates in the presence of purified TBC1D31 at the indicated concentrations. The schematic diagram is shown left. b) Mutant assays showing the effects of arginines within the GTPase‐activating protein (GAP) domain of TBC1D31 on the GTP hydrolysis activity for Rab22A in vitro. GTP hydrolysis was assayed in the reaction containing GTP‐loaded Rab22A (0.2 µM) in the presence of purified wide‐type (WT), individual mutant (individual transition from arginine [R] to lysine [K]) or the combined mutant TBC1D31 at a concentration of 3 µM. c) Rescue assays showing the effect of re‐introduction of WT or R430K mutant TBC1D31 on the co‐localization of EGFR and EEA1 or LAMP1 in *TBC1D31*‐knocked‐down HCCLM3 cells. Scale bar, 20 µm. d,e) Rescue assays showing the effect of re‐introduction of WT or R430K mutant TBC1D31 on the levels of EGFR on the cell membrane, as assessed by cell fractionation assays (d) and flow cytometry assays (e) in *TBC1D31*‐knocked‐down HCCLM3 cells. f) Rescue assays showing the effect of re‐introduction of WT or R430K mutant TBC1D31 on the EGFR accumulation and the activation of downstream cascades in *TBC1D31*‐knocked‐down HCCLM3 cells. g) Rescue assays showing the effect of re‐introduction of WT or R430K mutant TBC1D31 on the abilities of cell soft agar colony formation, migration, and invasion in *TBC1D31*‐knocked‐down HCCLM3 cells. Values are expressed as mean ± standard deviation (s.d.) of three or more independent replicates. *P* values were calculated using Student's *t* test unless specifically specified. ^*^, *P* < 0.05; ^**^, *P* < 0.01; ^***^, *P* < 0.001; n.s., not significant.

Next, we sought to investigate whether TBC1D31 plays a catalytic effect on Rab22A. To this end, we measured the release of inorganic phosphates using an in vitro continuous enzyme‐coupled optical assay to determine whether TBC1D31 mediates the single‐turnover kinetics of the hydrolysis of Rab22A loaded with GTP. Indeed, we observed that TBC1D31 enhances the GTP hydrolysis for Rab22A in a concentration‐dependent manner (**Figure** **6**a), suggesting its ability in transiting specific Rab GTPase(s) from an active GTP‐binding state to an inactive GDP‐binding state.

Then, we sought to identify the key amino acid residue(s) of TBC1D31 required for its catalytic activity. The key residue(s) for GAP‐mediated catalysis is the so‐called “arginine finger”, which is a highly conserved motif among GAP proteins.^[^ ^27^ ^]^ Within the GAP domain of TBC1D31, there exist six arginine residues, all of which are highly conserved among mammals (Figure 6b). Thus, we constructed six mutants by individual transition from the arginine (Arg, R) to lysine (Lys, K) and a combined mutant with all R‐to‐K transitions. We found that R479K and R585K show minimal effects, R435K, R471K, and R475K show mild effects, while the R430K and the combined mutant almost completely abolish the catalytic activity of TBC1D31 on GTP hydrolysis for Rab22A in vitro (Figure 6b). Thus, these findings suggest that the conserved Arg^430^ at GAP domain of TBC1D31 is required for its catalysis of GTP hydrolysis for Rab22A.

We further evaluated the functional relevance of Arg^430^ at TBC1D31 by re‐introduction of the wild‐type (WT) or R430K mutant TBC1D31 in TBC1D31‐knocked‐down HCC cells. The results showed that re‐introduction of WT TBC1D31 abolishes the inhibitory effects of *TBC1D31* knockdown on the co‐localization of EGFR with EEA1 or LAMP1, the levels of membrane‐bound EGFR and total EGFR, the activity of EGFR pathway, and the malignant phenotypes in TBC1D31‐knocked‐down HCCLM3 cells (Figure 6c–g). However, re‐introduction of the R430K mutant displayed no such rescue effects (Figure 6c–g). The similar results were also observed in TBC1D31‐knocked‐down Bel‐7402 cells (Figure S8, Supporting Information). Collectively, these findings suggest that Rab22A is a bona fide substrate for TBC1D31, and the TBC1D31‐Rab22A axis is responsible for reducing the endolysosomal trafficking of EGFR in HCC cells.

### 2.9 Downregulating TBC1D31 Overcomes the HCC Cells Resistance to Lenvatinib

**Figure 7:**
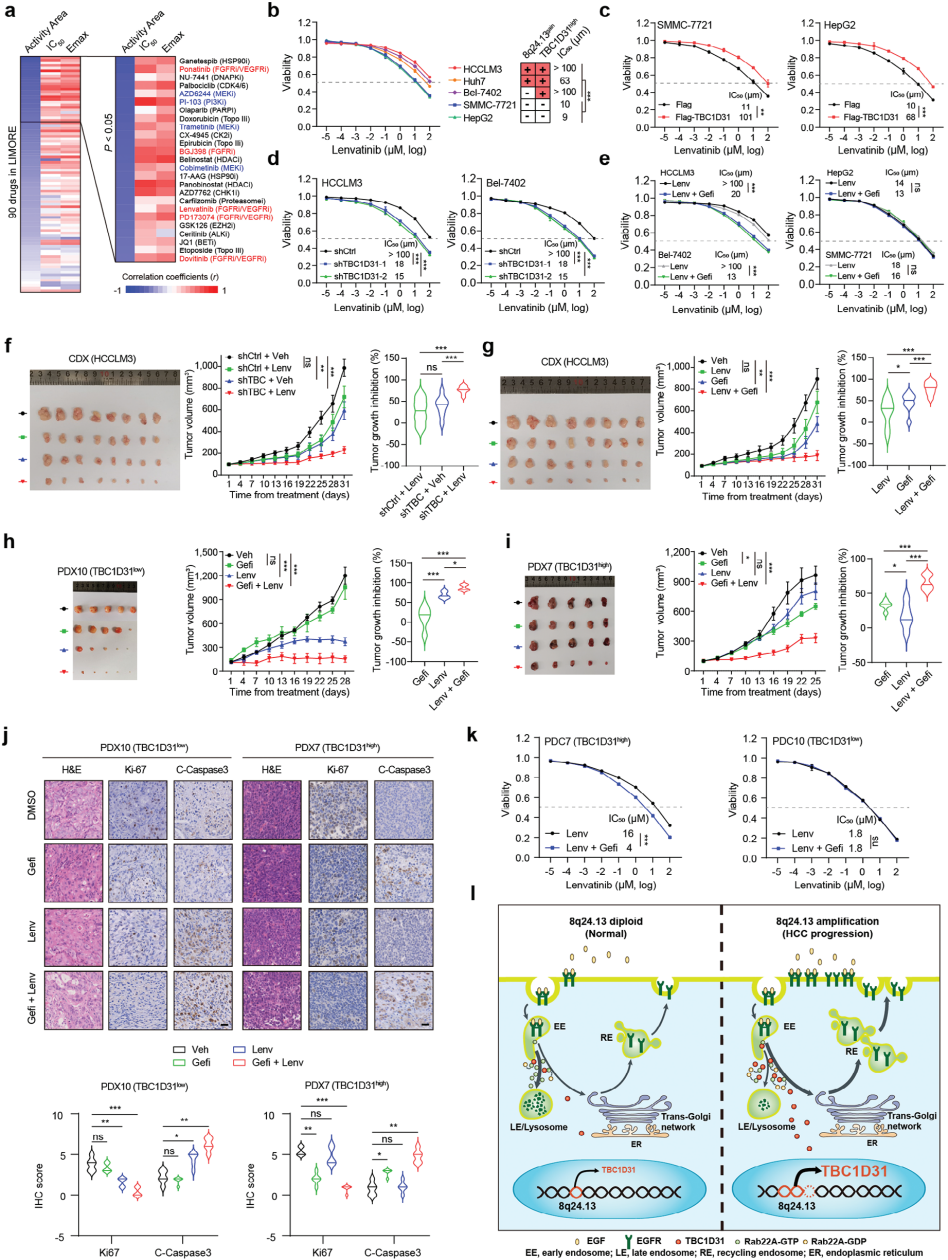
Downregulating *TBC1D31* sensitizes the HCC cells to lenvatinib treatment. a) Heatmap of the correlations between the TBC1D31 expression levels and compound sensitivities (including the activity area [AA], half‐maximal inhibitory concentration [IC_50_] and the maximum effect concentration [E_max_]) in 81 types of liver cancer cell line. The data of sensitivity of 90 compounds and gene expression levels were obtained from the Liver Cancer Model Repository (LIMORE) project. All 90 compounds were ranked according to the TBC1D31‐AA correlation coefficients. b) The cell viability of five types of HCC cell line after 72 h of lenvatinib treatment at the indicated concentrations, respectively. Right, the state of 8q24.13 gain, the state of TBC1D31 high expression and the IC_50_ for each cell lines. c) The effects of TBC1D31 overexpression on the sensitivities of SMCC‐7721 and HepG2 cells (with TBC1D31^low^) to lenvatinib. d) The effects of *TBC1D31* knockdown on the sensitivities of HCCLM3 and Bel‐7402 (with TBC1D31^high^) cells to lenvatinib. e) The effects of EGFR inhibition by gefitinib (2.5 µM) on the sensitivities of HCCLM3 and Bel‐7402 cells (with TBC1D31^high^; left panel) or SMCC‐7721 and HepG2 cells (with TBC1D31^low^) to lenvatinib. f) The effects of *TBC1D31* knockdown on the sensitivities of HCCLM3 cells to lenvatinib in the cell line‐derived xenografts (CDX) mice model. HCCLM3 cells stably expressing Tet‐On shCtrl or sh*TBC1D31* (equally pooled shTBC1D31‐1 and shTBC1D31‐2) were subcutaneously injected into the nude mice. After the tumor volume reaches ≈100 mm^3^, the xenografted mice begin to receive doxycycline (DOX) administration (2 mg mL⁻^1^, daily) and are randomized and orally administered with vehicle (0.5% CMC‐Na) or lenvatinib (4 mg k^−1^g) every 5 days (n = 8 mice/group). g) The effects of gefitinib‐induced (80 mg k^−1^g, every 5 days) EGFR inhibition on the sensitivities of HCCLM3 cells to lenvatinib treatment in the CDX mice model. h,i) Growth curves of PDX10 (h) and PDX7 (i) orally administered with vehicle (0.5% CMC‐Na), lenvatinib (4 mg k^−1^g), gefitinib (80 mg k^−1^g) or a drug combination in which each compound was administered at the same dose every 5 days (n = 5 mice/group). j) The IHC staining (left) and scores (right) of Ki67 and cleaved Caspases3 in tumor tissues from the PDXs. Scale bar, 200 µm. k) Cell viability determined by CCK‐8 assays for the TBC1D31^low^ patient‐derived primary tumor cells (PDC10) and the TBC1D31^high^ PDC7 upon the usage of lenvatinib or a drug combination with gefitinib (2.5 µM). l) The schematic model for the function and underlying mechanisms of TBC1D31 in HCC. Values are expressed as mean ± standard deviation (s.d.) of three or more independent replicates. *P* values were calculated using Student's *t* test unless specifically specified. ^*^, *P* < 0.05; ^**^, *P* < 0.01; ^***^, *P* < 0.001; n.s., not significant.

Through reanalyzing the pharmacogenomic data from 81 liver cancer cell lines generated by the Liver Cancer Model Repository (LIMORE) project,^[^ ^28^ ^]^ three MEK inhibitors (MEKi; including AZD6244, trametinib and cobimetinib) with their activities (including the activity area [AA], half‐maximal inhibitory concentration [IC_50_] and the maximum effect concentration [E_max_]) were found significantly associated with the expression levels of *TBC1D31* (**Figure** **7**a), which underscore the consistency of MEKi‐induced AKT phosphorylation in pancreatic cancers with high EGFR expression.^[^ ^29^ ^]^ Additionally, we observed significant correlations for five FGFR/VEGFR inhibitors (FGFRi/VEGFRi; including ponatinib, BGJ398, lenvatinib, PD173074 and dovitinib) (Figure 7a), with lenvatinib approved as a first‐line treatment for un‐resectable HCC patients. Although resistance to lenvatinib has emerged, it can be mediated through pathways involving EGFR.^[^ ^30^ ^]^

Therefore, we next focused on investigating whether the dysregulation of TBC1D31 confers distinct responses to lenvatinib in HCC cells. Cell viability assays showed that compared to HCC cells with low expression levels of *TBC1D31* accompanied by low EGFR levels/activities (e.g., HepG2 and SMMC‐7721), the *TBC1D31*‐amplified and/or ‐overexpressed HCC cells accompanied by high EGFR levels/activities (e.g., HCCLM3, Huh7 and Bel‐7402) exhibit a markedly increased resistance to lenvatinib (Figure 7b; Figure S9a, Supporting Information). Of note, lenvatinib treatment further enhanced the phosphorylation levels of EGFR in those cells with high TBC1D31 levels, suggesting that lenvatinib‐induced FGFR/VEGFR inhibition can parallelly activate the alternative signaling (e.g., EGFR pathway) (Figure S9a, Supporting Information). Furthermore, overexpression of TBC1D31 in HepG2 and SMMC‐7721 cells can induce increased resistance to lenvatinib (Figure 7c). Conversely, knockdown of *TBC1D31* sensitized HCCLM3 and Bel‐7402 cells to lenvatinib (Figure 7d). Meanwhile, we found that targeting EGFR by gefitinib also significantly increased the sensitivities of TBC1D31^high^ cells to lenvatinib, but not the TBC1D31^low^ cells (Figure 7e). We also replicated these findings in HCC cell line‐derived xenografts (CDX) models by using a doxycycline (DOX)‐inducible system to inhibit the expression of *TBC1D31* in HCCLM3 cells (Figure S9b, Supporting Information). Tumors from *TBC1D31*‐knocked‐down HCCLM3 cells were more sensitive to lenvatinib than those from control cells (Figure 7f; Figure S9c, Supporting Information). Accordingly, the tumor growth inhibition rates of the lenvatinib‐treated and *TBC1D31*‐knocked‐down groups were 25% and 47%, respectively, while the combined treatment group increased to 75% (Figure 7f). Consistently, EGFR inhibition by gefitinib also sensitized the HCCLM3‐derived CDXs to lenvatinib (Figure 7g; Figure S9d, Supporting Information).

These effects were further tested in HCC patient‐derived xenografts (PDX) models (Figure 7h–j). We established a total of 12 PDX models, among which Model 7 with *TBC1D31* amplification/high expression and EGFR high expression (designated as TBC1D31^high^) and Model 10 with *TBC1D31* non‐amplification/low expression and EGFR low expression (designated as TBC1D31^low^) were selected for testing (Figure S9e, Supporting Information). The TBC1D31^low^ PDX tumors were shown to be highly sensitive to lenvatinib, as concordantly evidenced by reduced Ki67 and increased cleaved Caspase3 (Figure 7h,j; Figure S9f, Supporting Information). As expected, gefitinib alone did not have a significant inhibitory effect on the growth of TBC1D31^low^ PDX tumors, and slightly enhanced the sensitivity of lenvatinib (Figure 7h; Figure S9f, Supporting Information). On the contrary, TBC1D31^high^ PDX tumors showed resistance to lenvatinib; however, treatment with gefitinib can remarkably potentiate their sensitivities to lenvatinib (Figure 7i,j; Figure S9g, Supporting Information). Moreover, we also assessed the effects of *TBC1D31* on sensitivity to lenvatinib in the patient‐derived primary tumor cells (PDCs) isolated from the both models. Knockdown of *TBC1D31* remarkedly reduced the malignant phenotypes and the expression and activity of EGFR in TBC1D31^high^ PDCs, while overexpression of TBC1D31 exhibited opposite effects in TBC1D31^low^ PDCs (Figure S9h–j, Supporting Information). Again, gefitinib treatment significantly increased the sensitivity of TBC1D31^high^ PDCs to lenvatinib; however, there is no such effect on TBC1D31^low^ PDCs (Figure 7k). Collectively, these results suggest that *TBC1D31*‐amplified HCC tumors possess resistance to lenvatinib. However, the vulnerability to inhibition of TBC1D31 and/or EGFR provides a promising therapeutic strategy to overcome this challenge.

## 3 Discussion

Although numerous studies have identified several common genomic CNAs in HCC, such as chromosome 1q gain, 8q gain, and 8p loss, the critical genes within them and their functional significance remain largely uncharacterized. Here, through the integrative analysis of large‐scale publicly available transcriptome data by using the ACE method, we identified for the first time that the genomic amplification at 8q24.13 is an independent prognostic indicator for HCC patients. Further, the functional and the mechanistic studies prioritized the *TBC1D31*, a member of the GTPase‐activating protein family, at the 8q24.13 amplification region being a critical oncogene involved in HCC development. Notably, 8q24 amplification is one of the most common genomic alterations in HCC, and is associated with HCC progression due to its ability to drive the oncogene *MYC* on 8q24.21.^[^ ^31^ ^]^ Thus, our findings highlight the biological relevance of the newly identified 8q24.13 amplification and its critical gene *TBC1D31* to the development of HCC in the context of broader chromosome 8q gain.

TBC GAPs have been found to regulate multiple cellular processes, including vesicle trafficking, cytoskeletal dynamics, and cell proliferation. They have been identified as important regulators of GTPase proteins in cancers. Several TBC GAP family members, e.g., TBC1D3, TBC1D7, TBC1D8, TBC1D10A, TBC1D16, TBC1D17, TBC1D23, EVI5, TRE2, and RN‐TRE, have been implicated in various types of cancer through their Rab‐GAP activity or GAP‐independent signaling.^[^ ^16^, ^32^, ^33^, ^34^, ^35^, ^36^, ^37^, ^38^, ^39^ ^]^ As an uncharacterized member of the TBC GAP family, TBC1D31 has recently been found to be a molecular scaffold for assembling OFD1, praja2 and PKA, and plays an important regulatory role in cilium biogenesis.^[^ ^40^ ^]^ However, its tumorigenic role remains unknown, especially its effect in modulating the GTPase signaling. We here demonstrated that TBC1D31 is a novel oncogene and functions through its Rab‐GAP activity.

Overactivation of the EGFR pathway is a common event in HCC. Apart from rare direct mutations or amplifications of EGFR, the activation mechanisms of the EGFR pathway are broadly categorized into ligand‐dependent (e.g., excessive production of the ligand EGF) and ligand‐independent pathways (e.g., crosstalk with other signaling pathways).^[^ ^41^ ^]^ Besides, emerging studies have revealed that the endocytic trafficking of EGFR is one of the vital cellular mechanisms in the spatial and temporal regulation of EGFR signaling.^[^ ^42^ ^]^ Several regulators have been shown to exert their actions by mediating the EGFR trafficking, such as Vav2, Sortilin, and FLCN.^[^ ^43^ ^]^ With respect to HCC, the Golgi membrane protein GOLM1 was shown to interact with EGFR and serves as a specific cargo adaptor to assist EGFR anchoring on the trans‐Golgi network (TGN) and recycling back to the cell membrane, without affecting the EGFR protein levels.^[^ ^15^ ^]^ The mechanism coordinating EGFR trafficking and its subsequent degradation in HCC cells remains poorly characterized. Here, we observed that TBC1D31 reduces the late endocytic trafficking of EGFR for lysosome‐mediated degradation. Further, consistency in clinical specimens indicates that the expression and activation of EGFR in HCC are driven, at least in part, by TBC1D31. Therefore, our data indicate another novel mechanism regulating the activation of EGFR pathway in HCC.

It was known that not all members of the TBC GAP family are enzymatically active, and only some of them can catalyze the GTPase activity of Rab. Here, we for the first time demonstrated that TBC1D31 has enzymatic activity against its bona fide substrate Rab22A. Rab22A was originally described as a Rab that could recruit EEA1^[^ ^44^ ^]^ and Rab5 guanine nucleotide exchange factor Rabex5.^[^ ^45^ ^]^ It is able to regulate the trafficking of recycling endosome containing clathrin‐dependent (EGFR, TfR, ATP7A, GLUT4 and etc.) and clathrin‐independent (MHC1, ITGB1, ADRB2 and etc.) endocytosed cargoes to the cell membrane in non‐melanocytes.^[^ ^46^ ^]^ Moreover, a recent study has revealed that Rab22A was required for the biogenesis of recycling endosomes through recruiting BLOC‐1 and BLOC‐2 to form a complex with KIF13A on endosomes.^[^ ^47^ ^]^ These findings therefore raise the hypothesis that Rab22A may serve as a distinct regulator of intracellular trafficking in a cell context‐dependent manner. We hereby demonstrate that Rab22A, as the major substrate of TBC1D31, potentiates the endolysosomal trafficking of EGFR. This is in concordance with the role of Rab22B (also known as Rab31) in epidermoid carcinoma cells,^[^ ^25^ ^]^ suggesting the convergent function of Rab22 subfamily. Together, our results provide an important supplement for the understanding of the function of Rabs in endolysosomal trafficking of specific cargoes, e.g., EGFR.

Lenvatinib (a FGFRi/VEGFRi) has recently been proven to be non‐inferiority and can improve clinical efficacy in the treatment of HCC; however, the primary and acquired resistance of lenvatinib limits its wider clinical application.^[^ ^30^, ^48^ ^]^ The mechanism of resistance to lenvatinib may be complex and multifactorial, mainly due to abnormal activation of other RTKs such as EGFR and IGFR.^[^ ^30^ ^]^ These feedback systems downstream of RTKs, such as the MAPK/ERK1/2 or PAK2‐ERK5 signaling axis, enable cancer cells to receive common survival signals through cross‐talk, leading to drug resistance.^[^ ^30^, ^48^ ^]^ Indeed, an increased EGFR activation was observed in lenvatinib‐treated HCC cells, especially in those *TBC1D31*‐amplified ones (Figure S9a, Supporting Information). Moreover, the *TBC1D31*‐amplified HCC tumors with the MAPK/ERK1/2 and PI3K/AKT hyperactivated by the TBC1D31/Rab22A/EGFR axis exhibited primary resistance to lenvatinib (Figure 7b). However, this resistance can be overcome through TBC1D31 inhibition (by knockdown), or EGFR inhibition (by gefitinib), or their combined inhibition, which reduces the dependence of *TBC1D31*‐amplified HCC cells on the EGFR‐driven MAPK/ERK1/2 and PI3K/AKT pathways. Given the high frequency of genomic gain of *TBC1D31* in HCCs (≈50%), our findings thereby provide an indicator of primary resistance to lenvatinib, and suggest a potential therapy strategy that simultaneously inhibits the TBC1D31‐EGFR axis when using lenvatinib in those HCC cases with *TBC1D31* amplification. This strategy may also be promise for the treatment of other types of cancer characterized by frequent amplification of *TBC1D31* (> 50%; Figure S1d, Supporting Information), such as uveal melanoma, ovarian cancer, and uterine carcinosarcoma. Certainly, extensive clinical research and trials are needed to evaluate the effectiveness and safety of this strategy. Additionally, recent studies have demonstrated that EGFR activation leads to an immunosuppressive microenvironment, and tumors with highly active EGFR exhibit reduced response to immune checkpoint inhibitors (ICIs).^[^ ^49^ ^]^ Therefore, the combination of targeted therapy and immunotherapy is also worth exploring in the context of TBC1D31. In addition, the pharmacogenomic analysis here also revealed the associations of TBC1D31 with other targeted inhibitors (Figure 7a), indicating the possibility of exploring other alternative combination therapy strategies.

## 4 Conclusion

In conclusion, our findings demonstrate that the 8q24.13 amplification‐driven upregulation of TBC1D31 enhances its catalytic effect on Rab22A, thereby reducing the endolysosomal trafficking of EGFR for degradation, ultimately promoting the EGFR signaling and tumorigenesis in HCC cells (Figure 7l). These results present a promising therapeutic opportunity to target the TBC1D31‐EGFR axis. Furthermore, beyond the 8q24.13 amplification, other genomic CNAs may also have potential oncogenic effects, which deserve further in‐depth investigation.

## 5 Experimental Section

### Human HCC Tumor Tissues

The discovery stage includes an in‐house cohort (designated as DISC1) and five publicly available cohorts (designated as DISC2 – 6), totally consisting of 814 patients with HCC. The DISC1 consists of 31 HCC patients who were recruited from the First Affiliated Hospital of Zhejiang University (Hangzhou City, China) and Beijing Cancer Hospital (Beijing, China) from 2007 to 2010. The gene expression profile datasets of tumor tissues and non‐tumor liver tissues from the five publicly available cohorts were obtained from the Gene Expression Omnibus (GEO): GSE25097 (DISC2; n = 243), GSE22058 (DISC3; n = 96), GSE14520 (DISC4; n = 208), and GSE36376 (DISC5; n = 123), and the Cancer Genome Atlas (TCGA; https://portal.gdc.cancer.gov/) data portal (DISC6; n = 113) (Table S1, Supporting Information). In the validation stage, a cohort consisting of 212 patients with HCC (designated as VALI cohort) was recruited from the Jinling Hospital (Nanjing City, China) and Jindu Hospital (Nanjing City, China) between 2007 and 2013. All the HCC patients recruited in this study were newly diagnosed, previously untreated (chemotherapy or radiotherapy), pathologically confirmed, and proved not to have other types of cancer. Each tissue specimen was reviewed by a board‐certified pathologist to confirm that the frozen section was histologically consistent with tumor or non‐tumor liver tissues, and the tumor sections had to contain more than 70% tumor cell nuclei and less than 20% necrosis. The written informed consent was obtained from each patient, and the demographic and clinical data were collected by structured questionnaire. This study was approved (Approval number: AF/SC‐08/02.81) by the Medical Ethical Committees of Beijing Institute of Radiation Medicine (Beijing, China). The detailed clinical characteristics of these HCC patients are described in Table S1 (Supporting Information). The RNAs extracted from the tumor tissues and non‐tumor liver tissues of the patients from the DISC1 cohort were used for gene expression profiling, and the matched genomic DNAs were used for genome‐wide SNP genotyping by Affymetrix SNP Array 6.0. The genomic DNAs from the tumor tissues and non‐tumor liver tissues of all patients from the VALI cohort were used for quantifying the genomic copy number of 8q24.13 locus. Additionally, among the 212 subjects in the VALI cohort, 168 ones with tissues are available for tissue microarray (TMA) construction. Thus, the tissues from these 168 patients were used for TMA construction and subsequent immunohistochemistry (IHC) assays.

### Cell Lines

Several types of HCC cell lines, including HepG2, Bel‐7402, SMMC‐7721, Huh7 and HCCLM3, and HEK293T were obtained from the China Center for Type Culture Collection (CCTCC; Wuhan City, China). These cell lines were maintained in Dulbecco's modified Eagle's medium supplemented with 10% heat‐inactivated fetal bovine serum and 1% penicillin and streptomycin. The cells were incubated at 37 °C in a humidified incubator containing 5% CO_2_.

### Detection of CNAs by ACE Based on Transcriptomic Data

The Analysis of CNAs by Expression data (ACE) method was employed to the normalized gene expression datasets of HCC tumor tissues and non‐tumor liver tissues from the six discovery cohorts according to the instruction described in a previous study.^[^ ^9^ ^]^ This algorithm suppresses individual gene‐specific expression patterns by summing the distance‐weighted neighborhood scores (NSs) of neighboring genes over genomic regions and emphasizes the signal of CNAs. For the GEO gene expression profile datasets generated by microarrays (i.e., from the DISC1, 2, 3, 4 and 5), we only retained the probes that were available in more than 90% of the samples for further analysis. Expression levels were calculated based on the average intensity of probes that are annotated as exonic ones. For the TCGA RNA sequencing (RNA‐seq) dataset (i.e., from DISC6), the fragments per kilobase of exon model per million reads mapped (FPKM) values were subjected to log_2_ transformation. All the expression data were normalized by quantile normalization for further analyses. For detection of significant CNAs in HCC, paired *t* statistic was used to compute the NSs by comparing the signal in the tumor group relative to the non‐tumor group. For detection of significant CNAs in a single sample, z‐score was used to compute the NSs. The statistical significance of the NS is estimated by 1000 sample labels permutation and 1000 gene names permutation, and the segments harboring at least 10 aberrant NSs with false discovery rate (FDR) < 0.001 were determined as potential recurrent CNAs.

### Detection of CNAs Based on Genomic Genotyping Data

*In‐house dataset of the DISC1 cohort*. Genomic DNAs of each individual from the DISC1 cohort (Table S1, Supporting Information) were amplified and hybridized onto an Affymetrix Genome‐Wide Human SNP Array 6.0 by an Affymetrix service facility (CapitalBio, China) according to the manufacturer's instructions (Affymetrix, USA). The amplified DNAs were then fragmented, labeled, and hybridized to the arrays. The arrays were then washed, scanned, and the image data were analyzed. For the raw. CEL file, CRMA method was used to pre‐process the probe signal intensities. The procedure included that signal intensities are calibrated for offset and crosstalk between alleles, and normalized for probe sequence effects. After summarizing to probe‐set level signals, the fragment‐length effects were normalized. The raw copy numbers were calculated by paired analysis, taking paired tumor‐adjacent tissue as a reference for every tumor tissue. Then, the segmented copy number profiles were analyzed using the Circular Binary Segmentation (CBS) algorithm with default parameters in the R package “DNA‐copy”.^[^ ^50^ ^]^

### Publicly Available Genomic Datasets of the Discovery Cohorts

Two genomic genotyping datasets of the discovery cohorts (DISC4 and DISC6; Table S1, Supporting Information) were obtained from the TCGA data portal (Mar 31, 2015; genotyped by Affymetrix SNP 6.0 arrays) and the GEO database (Accession No. GSE14322; genotyped by CGH arrays), respectively. Additionally, the frequencies of 8q24.13 genomic alterations across multiple types of cancer were obtained from TCGA data portal (Mar 31, 2015). Changes of Log ratio (LR) segment mean value > 0.3 or < −0.3 were defined as the copy number gain or loss, respectively. CNAs were visualized from the individual representational oligonucleotide microarray analysis plots of the specific HCC samples using the Integrated Genomics Viewer (IGV) software.^[^ ^51^ ^]^

### CNA Genotyping by qPCR Assays

Total DNA from the frozen tissues of all patients from the VALI cohort (n = 212) or liver cell lines were extracted using Trizol Reagent (Invitrogen, USA) according to the manufacturer's instructions. To determine the DNA copy numbers at chromosome 8q24.13 locus, three pairs of primers were designed on the basis of the intron sequences of genes at this locus, including *C8orf76*, *ATAD2* and *NDUFB9*. The average genomic content of three genes, including *LTBP1* (at 2p22.2 locus), *SATB1* (3p24.3) and *ANO3* (11p14.3), which were confirmed to having no CNAs in our HCC cohorts (data not shown), was used as the internal reference. Primers were designed using the software Primer3.^[^ ^52^ ^]^ The real‐time quantitative PCR (qPCR) assays were performed using the SYBR Green Universal PCR Master Mix (KR0389‐v8.12, KAPA, USA) on the iQ5 Real‐Time PCR System (Bio‐Rad, USA) according to the manufacturer's protocol. The genomic region with relative copy number greater than 1.25 was defined as a genomic gain. Primer sequences are listed in Table S10 (Supporting Information).

### qRT‐PCR Assays

Total RNAs from the frozen tissues and cell lines were extracted using Trizol Reagent (Invitrogen, USA) according to the manufacturer's instructions. Their quality and integrity were measured using a spectrophotometer (Nano Vue, GE, USA) and agarose gel electrophoresis. The quantitative reverse transcription PCR (qRT‐PCR) assays were performed following the reverse transcription by SuperScript first‐strand synthesis kit (Takara, Japan). The *β‐actin* (*ACTB*) gene was used as the internal reference for normalization. Primer sequences are listed in Table S10 (Supporting Information).

### siRNAs, shRNAs and Plasmids

The small interfering RNAs (siRNAs) targeting *TBC1D31*, *EGFR* or *Rab22A*, and a non‐targeting control siRNA were synthesized by RiboBio Co., (Guangzhou City, China). The cells were transfected twice with 50 nM siRNAs using riboFECT (RiboBio, Guangzhou City, China) according to the manufacturer's instructions. Lentiviral short hairpin RNA (shRNA) constructs (pLV‐puro‐Luciferase) coding a scramble sequence and two independent sequences targeting to *TBC1D31* were obtained from Hanbio Co., (Shanghai, China). Lentivirus packing expression vector (pLV‐Neo‐Flag‐TBC1D31) was constructed by Inovogen (Beijing, China). To generate the stable knockdown or overexpression cell lines, HCC cell lines were transfected with the indicated lentiviruses (multiplicity of infection [MOI] = 10 ∼ 20). The stable clones were selected using 2 µg/mL puromycin or 500 µg/mL G418 (Sigma, USA). Real‐time qRT‐PCR or immunoblotting assays were performed to determine the knockdown or overexpression efficiency, respectively. The cDNAs of *Rab4A*, *Rab5A*, *Rab7A*, *Rab11A* and *Rab22A* were kindly provided by Prof. Jian Wang (at the State Key Laboratory of Medical Proteomics, Beijing, China). The cDNAs of *Rab21A* were synthesized by Inovogen (Beijing, China). These cDNAs were used as templates and amplified using specific primers. Further, amplified PCR products were cloned into the pCMV‐Myc plasmid for ectopic expression in cells. All constructs used in this study were confirmed by DNA Sanger‐sequencing. The sequences of all shRNAs and siRNAs are listed in Table S10 (Supporting Information).

### High‐Content Function Screening Assays

To evaluate the tumorigenic roles of the 12 candidate genes within 8q24.13 locus, the HCS assays were performed in HepG2 and SMMC‐7721 cells. For each gene, four individual shRNAs (pLV‐puro‐EGFP) were designed and pooled to minimize the possibility of off‐target effects. All the shRNAs were purchased from Genechem (Shanghai, China), and the sequences were listed in Table S10 (Supporting Information). The HepG2 or SMMC‐7721 cells were infected by shRNA‐expressing lentiviruses that targeted each gene separately. After 48 – 72 hours (h), cells were used for subsequent assays. Each experiment was repeated three times and reported as three independent replicates. For cell number counting assays, cells (2 × 10^3^ cells/well) were seeded onto the 96‐well plates. The total number of cells (EGFP‐labeled) in the same field was measured at 0, 24, 48, 72 and 96 h using the Thermo Scientific Cellomics ArrayScan VTI platform (USA). For wound healing assays, cells were plated in 96‐well plates (2 × 10^4^ cells/well). When the cells reached 90% confluence, a scratch was made, and the detached cells were removed by washing using the culture medium. Phase contrast images were obtained in the same field at 0 and 24 h using the Thermo Scientific Cellomics ArrayScan VTI platform (USA).

### Cell Growth Assays

*Cell Counting Kit‐8 (CCK‐8) assays*. The CCK‐8 (Dojindo, Japan) assays were used to measure the cell proliferation. Cells were trypsinized and counted by using a handheld cell counter (Millipore, Germany). A total of 2 ∼ 3 × 10^3^ cells per well were seeded onto the 96‐well plates and incubated for 1, 2, 3, 4, 5 and 6 days, respectively. The cells were then incubated with the CCK‐8 reagent for 1 h prior to measure the absorbance of 450 nm using an enzyme‐linked immunosorbent assay (ELISA) plate reader (Thermo, USA). Each experiment was consisted of four replications and at least three individual experiments were carried out. For plate colony formation assays, a total of 1 × 10^3^ cells (for assessing the pro‐tumoral effects in overexpression assays) or 2 × 10^3^ cells (for assessing the anti‐tumoral effects in knockdown assays) were seeded in 6‐cm plates. After 2–4 weeks incubation, the colonies were washed with PBS and stained with 0.5% crystal violet‐methanol for 5 minutes (min) at room temperature. The colonies were scanned and counted. A mean number of colonies was obtained from three independent experiments. For soft agar colony formation assays, the base agar was prepared to 42 °C (1.2% agarose:2 × DMEM = 1:1) and added to 6‐well dish (2 mL/dish), allowed to solidify. The top agar was cooled to 37 °C in water bath with 2 × DMEM in the same manner as the base agar (0.7% agarose:2 × DMEM = 1:1). Then, the cells were counted and added in the top agar. The mixture (1 mL) was added to 6‐well dish (1 × 10^3^ cells/mL/dish for assessing the pro‐tumoral effects in overexpression assays, or 2 × 10^3^ cells/mL/dish for assessing the anti‐tumoral effects in knockdown assays), allowed to solidify. After 3–4 weeks incubation, the colonies were scanned and counted. A mean number of colonies was obtained from three independent experiments.

### Cell Migration and Invasion Assays

For migration and invasion assays, a total of 7 × 10^4^ cells (200 µL; for assessing the pro‐tumoral effects in overexpression assays) or 1.4 × 10^5^ cells (200 µL; for assessing the anti‐tumoral effects in knockdown assays) were planted on the top chamber of each insert (Cat. 353097, BD Biosciences, USA) or matrigel invasion chamber (Cat. 354480, BD Biosciences, USA). Then, 800 µL DMEM supplemented with 20% fetal bovine serum (FBS) was injected into the lower chambers. After incubation at 37 °C for 24 h for migration and 48 h for invasion, respectively, the cells adhering to the lower side of the inserts were stained with 0.5% crystal violet solution, and then imaged and counted by IX71 inverted microscope (Olympus, Japan).

### Nude Mice Studies

Five‐ to six‐week‐old male nude BALB/c mice were purchased from the Vital River Laboratories (VRL, Beijing, China). All mice were maintained in a specific‐pathogen‐free (SPF) facility, and all the related protocols were performed in accordance with a protocol approved (Approval number: IACUC‐20210223‐09MTL) by the Animal Ethics Committee of Beijing Institute of Radiation Medicine (Beijing, China). In the subcutaneous implantation mice model, the HCCLM3 cells transfected with shCtrl or sh*TBC1D31* (1 × 10^6^ cells diluted in 100 µL PBS), and the Bel7402 cells transfected with Flag or Flag‐TBC1D31 (8 × 10^5^ cells diluted in 100 µL PBS) were grafted subcutaneously in each side of the mice back (n = 8 – 10). Tumor volumes were estimated every three days from two‐dimensional caliper measurements using the equation V = (1/2) × L × W^2^, where V = volume (mm^3^), L = length (mm), and W = width (mm), and reported as volume mean ± standard deviation (s.d.) for each mouse group. When the tumors reached a maximum of 1500 mm^3^, mice were euthanized and the tumors were harvested and procured for immunohistochemistry (IHC) assays. The following antibodies were used for IHC assays: anti‐EGFR (1:300; #4267, Cell Signaling, USA), anti‐phosphorylated‐EGFR (p‐EGFR, Tyr1068; 1:300; #3777, Cell Signaling, USA), anti‐phosphorylated‐AKT (p‐AKT, Ser473; 1:300; ab81283, Abcam, USA), anti‐phosphorylated‐ERK1/2 (p‐ERK1/2, Thr202/Tyr204; 1:300; #9101, Cell Signaling, USA) and anti‐Ki‐67 (1:200; sc‐23900, Santa Cruz, USA). In the tail vein injection model, a total of 1.25 × 10^6^ luciferase‐tagged HCCLM3 cells with or without *TBC1D31* knockdown (diluted in 250 µL PBS) were injected into the tail vein of the mice (10 – 11 mice/group). The mice were monitored once a week using a bioluminescence imaging. After 4 weeks, the mice were sacrificed, and their livers, lungs and brains were harvested, fixed in 4% paraformaldehyde, and embedded in paraffin. The sections were stained with hematoxylin and eosin (H&E) and the number of lung metastases was calculated independently by two pathologists.

### Immunoblotting Assays

Cells were harvested in PBS (4 °C) and cell pellets were lysed using NETN buffer containing complete protease inhibitor cocktail (Roche, Germany) and phosphatase inhibitor cocktail (Cwbiotech, China). Protein lysates were then subjected to SDS‐PAGE and Western blotting assays were performed using the antibodies specific to TBC1D31 (HPA023710, Sigma, USA), Rab22A (12125‐1‐AP, Proteintech, USA), EGFR (#4267, Cell Signaling, USA), p‐EGFR (Tyr1068; #3777, Cell Signaling, USA), AKT (60203‐1‐Ig, Proteintech, USA), p‐AKT (Ser473; ab81283, Abcam, USA), ERK1/2 MAPK (#4695, Cell Signaling, USA), p‐ERK1/2 MAPK (Thr202/Tyr204; #9101, Cell Signaling, USA), JNK (66210‐1‐Ig, Proteintech, USA), phosphoylated‐JNK (p‐JNK, Thr183/Tyr185; #4671, Cell Signaling, USA), p38 MAPK (#9212, Cell Signaling, USA), phosphoylated‐p38 MAPK (p‐p38, Thr180/Tyr182; #9215, Cell Signaling, USA), EEA1 (610456, BD Biosciences, USA), LAMP1 (ab24170, Abcam, USA) and Flag (F3165, Sigma, USA), respectively. GAPDH (CW0100, Cwbiotech, China) was used as loading control. Densitometric analyses of the immunoblot bands were performed by using Image J (v1.53).

### Immunohistochemistry Assays

A total of 168 pairs of formalin‐fixed, paraffin‐embedded HCC tissues and adjacent non‐tumor liver tissues from the HCC patients of the VALI cohort were used for immunohistochemistry (IHC) analyses. The clinical information of these HCC patients is provided in Table S1 (Supporting Information). Tissue microarrays (TMAs) were constructed using the automated tissue arrayer (ATA‐27; Beecher Instruments, USA). IHC staining was performed using the monoclonal antibody against TBC1D31 (1:200; HPA023710, Sigma, USA), EGFR (1:1000; #4267, Cell Signaling, USA), p‐EGFR (Tyr1068; 1:300; #3777, Cell Signaling, USA), p‐AKT (Ser473; 1:300; ab81283, Abcam, USA), p‐ERK1/2 MAPK (Thr202/Tyr204; 1:300; #9101, Cell Signaling, USA), cleaved Caspase‐3 (1:200; GB11532‐100, Servicebio, China) or Ki‐67 (1:200; sc‐23900, Santa Cruz, USA) on TMA sections after antigen retrieval. The signal was visualized after incubating with 3, 3′‐diaminobenzidine and counterstaining with hematoxylin (Sigma‐Aldrich, USA). Briefly, the protein expression levels were determined semi‐quantitatively according to the percentage of positively stained cells and the staining intensity as previously described.^[^ ^53^ ^]^ Briefly, a proportion score was assigned representing the estimated proportion of positive staining tumor cells (0, none; 1, < 1/100; 2, 1/100 to < 1/10; 3, 1/10 to < 1/3; 4, 1/3 – 2/3; and 5, > 2/3). The average estimated intensity of staining in positive cells was assigned an intensity score (0, none; 1, +; 2, ++; 3, +++; and 4, ++++). The overall scores (0 or 2 – 9) were obtained by combining these two parameters.

### Correlation‐Based Gene Set Enrichment Analyses

To explore the potential pathways which were affected by TBC1D31, we performed correlation‐based gene set enrichment analyses (GSEA) on the basis of the mRNA expression profile data sets from four discovery cohorts (DISC1, DISC2, DISC3 and DISC4). The Pearson correlation coefficients between the expression levels of TBC1D31 and each of those other genes were computed and then ranked. Then, the rank list was used to identify the significantly enriched gene sets based on MsigDB 4.0 by GSEA.^[^ ^54^ ^]^ The significance was assessed by 1000 permutations with gene labels sampling. Gene set with the false discovery rate (FDR) < 0.01 was considered to be statistically significant.

### TBC1D31 Co‐Dependency Analyses

The Project Achilles RNAi viability database was used to identify the gene dependencies that are most closely associated with TBC1D31 dependency. This analysis was performed on the publicly available Achilles cell lines, comprised of 17309 gene dependency z‐score across 712 cancer cell lines.^[^ ^55^ ^]^ Because TBC1D31 dependency was only measured in a subset of 600 cell lines, only this subset was analyzed. Gene dependencies were ranked by Spearman correlation coefficients with TBC1D31 dependency across 600 cancer cell lines, and FDR (< 0.05) was controlled by the Benjamin and Hochberg method. The pre‐ranked GSEA was performed to identify the signaling pathways positively correlated with the TBC1D31 dependency based on MsigDB (v6.0, c2) by using R package “clusterProfiler”.^[^ ^56^ ^]^

### Flow Cytometry Assays

Cells were treated with serum‐free medium and starved overnight. Cells were treated with 10 ng/mL EGF for the indicated time at 37 °C. EGF uptake was halted by transferring the cells on ice. Then, the cells were washed with ice‐cold phosphate‐buffered saline PH 4.0 (cold acid buffer) twice to remove the membrane‐bound EGF and halt EGFR internalization. Cells were cultured in DMEM without serum in an incubator at 37 °C and 5% CO_2_ for the indicated time to observe the EGFR recycling process. Cells were harvested in PBS and washed with cell staining buffer (Cat#420201, Biolegend, USA). A total of 2.5 × 10^5^ cells in each tube were centrifuged at 350 g for 1 min to remove the supernatant, and followed by incubation with 20 µL of Clear Back (human Fc receptor blocking regent) for 5 min at room temperature. Then, the cells were incubated with Alexa 488 anti‐human EGFR antibody (1:100; Cat#352908, Biolegend, USA) in cell staining buffer for 20 min on ice. After being washed twice with cell staining buffer, cells were fixed with 4% paraformaldehyde at room temperature for 10 min. Then, the fluorescence intensity was examined by flow cytometry using a FACS Calibur system (BD Biosciences, USA) and expressed as mean fluorescence intensity (MFI) ± standard deviation (s.d.).

### Cell Fractionation Assays

Preparation of cytoplasmic and membrane fractions was performed using the Cell Fractionation Kit (Cat#9038, Cell Signaling Technology, USA) according to the manufacturer's instructions. Briefly, cells were collected and washed with PBS by centrifugation at 350 × g for 5 min. Cell pellets were subsequently re‐suspended in CIB buffer and incubated on ice for 5 min. After centrifugation at 500 × g for 5 min, the supernatant was collected as cytoplasmic fraction. The pellet was then recovered and re‐suspended in MIB buffer. The soluble fraction containing the membrane and organelle components was obtained as membrane fraction and for further analyses.

### Immunoprecipitation Assays

Cells were lysed using the NETN lysis buffer (100 mM NaCl, 1 mM EDTA, Tris, pH 8.0, 0.5% NP‐40 and complete protease inhibitor cocktail (Roche, Germany)). After incubation for 20 min on ice, the lysates were centrifugated at 12,000 rpm for 10 min. Agarose A/G beads (sc‐2003, Santa Cruz, USA) were incubated with the indicated antibodies (1 – 2 ug) for 4 h at 4 °C with rotation. The pre‐treated beads were added in 1 – 5 mg of clarified protein lysate at 4 °C with rotation. The beads‐bound proteins were washed three times with 1 mL NETN lysis buffer at 4 °C. Then, the 2× loading buffer was added, and the samples were boiled at 95 °C for 10 min, spun to pellet the beads and subjected to SDS‐PAGE. The antibodies for immunoprecipitation assays include the anti‐rabbit Rab22A (12125‐1‐AP, Proteintech, USA), anti‐mouse Flag (F3165, Sigma, USA), goat anti‐rabbit IgG (CW0103, Cwbiotech, China) and goat anti‐mouse IgG (CW0102, Cwbiotech, China).

### GST Pull‐Down and SFB Pull‐Down Assays

The coding sequence of *Rab22A* (NM_020673.3) was subcloned into GST vector (pGEX‐4T‐2). The GST vector and GST‐Rab22A vector, respectively, were transformed into BL21 *E*. Coli cells. Cells were then induced with 1 mM IPTG at 37 °C for 4 h at OD600 of 0.6, and were re‐suspended in lysis buffer (300 mM NaCl, 20 mM Tris‐HCl, 1% Triton X‐100, and 1 µg/mL aprotinin and leupeptin) and sonicated on ice. The cell lysates were cleared by centrifugation at 12000 × rpm for 10 min at 4 °C and were incubated with Glutathione‐Sepharose resins for 1 h at 4 °C. The beads with GST‐tag protein were washed with lysis buffer three times. The bound proteins were used for pull‐down assays. The coding sequences of wild‐type *TBC1D31* (NM_145647.4) was subcloned into the SFB‐tagged (S‐protein tag, Flag epitope tag, and streptavidin‐binding peptide tag) vector. SFB‐TBC1D31 was transfected into HEK293T cells. Cells were lysed with NETN buffer (100 mM NaCl, 1 mM EDTA, 20 mM Tris‐HCl pH 8.0, and 0.5% Nonidet P‐40) containing protease inhibitors at 4 °C for 30 min. The cell lysates were cleared by centrifugation at 12000 × rpm for 10 min at 4 °C and were incubated with streptavidin‐conjugated beads (GE Healthcare, USA) for 2 h at 4 °C with gentle rocking. The beads with SFB‐TBC1D31 were washed three times with NETN buffer and were eluted with 1 mg/mL biotin (Sigma, USA) for 1 h at 4 °C. The bound proteins were used for pull‐down assays. For in vitro GST pull‐down assays, SFB‐TBC1D31 was incubated with GST or GST‐Rab22A in NETN buffer for 3 h at 4 °C. The beads‐bound proteins were then washed five times with NETN buffer and resolved on SDS‐PAGE. SFB‐TBC1D31 was detected by Flag antibody. For in vitro SFB pull‐down assays, the beads with GST‐Rab22A were eluted with GST elution buffer (10 mM L‐Glutathione reduced in 10% Tris‐HCl pH 8.8; Sigma, USA) for 1 h at 4 °C. The streptavidin‐conjugated beads bound with SFB or SFB‐TBC1D31 were incubated with GST‐Rab22A in NETN buffer for 3 h at 4 °C. The beads‐bound proteins were then washed five times with NETN buffer and resolved on SDS‐PAGE. SFB‐TBC1D31 was detected by GST antibody (1:1000; ab92, Abcam, USA).

### GTPase‐Activating Protein Activity Assays

The GST‐Rab22A recombinant protein was purified from *E. coli*, and Flag‐TBC1D31 protein was purified from HEK293T cells. Rab22A GTPases were loaded with GTP by incubating 2 – 3 mg of protein with a 25‐fold molar excess of GTP at 25 °C for 1 h in buffer (20 mM HEPES pH7.5, 150 mM NaCl, 5 mM EDTA). Free nucleotide was then removed with a D‐Salt column (Pierce Biotechnology, USA). The single‐turnover kinetics of intrinsic and GTPase‐activating protein (GAP)‐accelerated GTP hydrolysis was measured by a continuous enzyme‐coupled optical assay for the release of inorganic phosphate, with the use of reagents from the EnzChek Phosphate Assay Kit (Thermo Fisher). GAP assays were then conducted in 1 × assay buffer (20 mM HEPES pH 7.5, 150 mM NaCl, 0.15 mM 2‐amino‐6‐mercapto‐7‐methylpurine ribonucleoside, 0.75 U/mL purine nucleoside phosphorylase, 10 mM MgCl_2_) containing 0.2 µM Rab22A‐GTP and increasing concentrations of TBC1D31. The absorbance at 360 nm was monitored using an ELISA plate reader (Thermo, USA).

### Immunofluorescence Assays

HCCLM3 cells (1 × 10^5^) were seeded on 20 mm glass bottom dishes. Then, the cells were treated with EGF (10 ng/mL) for indicated time after serum starvation for 8 h. The cells were then fixed in 4% paraformaldehyde (PFA) and permeabilized with PBS containing 0.5% Triton X‐100. Blocking solution (5% milk/PBS) was applied for 10 min followed by incubation with mouse monoclonal Rab22A antibody (1:50 in 5% horse serum/PBS solution; sc‐390726, Santa Cruz, USA), rabbit anti‐TBC1D31 antibody (1:100 in 5% horse serum/PBS solution; HPA‐023710, Sigma, USA), rabbit anti‐EGFR antibody (1:400 in 5% horse serum/PBS solution; #4267, Cell Signaling, USA), mouse monoclonal EEA1 antibody (1:500 in 5% horse serum/PBS solution; 610456, BD Biosciences, USA) or mouse monoclonal EGFR antibody (1:500 in 5% horse serum/PBS solution; ab30, Abcam, USA), rabbit anti‐LAMP1 antibody (1:500 in 5% horse serum/PBS solution; ab24170, Abcam, USA) for 20 min at room temperature. Cells were washed three times with PBS and incubated with secondary anti‐rabbit Rhodamine Red‐X (111‐295‐144, Jackson, USA) and anti‐mouse FITC (115‐095‐146, Jackson, USA) antibodies (1:1000 or 1:200 in 5% horse serum/PBS solution) for 20 min at room temperature. Cells were washed three times with PBS and incubated with DAPI (Invitrogen, USA) to visualize nuclear DNA for 1 min at room temperature. Cells fixed at different indicated times were imaged using a confocal laser scanning microscope (Nikon, Japan) equipped with a Plan Fluor 60 × oil objective lens after co‐staining with antibodies against EGFR and EEA1 or LAMP1. EGFR‐EEA1 or EGFR‐LAMP1 co‐localization was analyzed by Manders method of pixel intensity correlation measurements using the Image J/Fiji‐Coloc2 plugin (n = 50 cells for each time point from three independent experiments).

### In Silico Pharmacogenomics Analyses

The drug sensitivity datasets of liver cancer cell lines were obtained from the LIMORE,^[^ ^28^ ^]^ which include 31 liver cancer cell models and 90 single drugs, and the corresponding RNA‐seq raw files of the 81 liver cancer cell models were downloaded from the Sequence Read Archive (SRA, ID: SRP102549). The levels of *TBC1D31* in those 81 liver cancer cell models were calculated based on the RNA‐seq data. The correlations between the drug sensitivity values (including the activity area [AA], half‐maximal inhibitory concentration [IC_50_] and the maximum effect concentration [E_max_]) and the *TBC1D31* expression levels were evaluated using Spearman correlation analysis, and *P* < 0.05 was considered to be statistically significant.

### Drug Sensitivity Testing

*In vitro assays in HCC cell lines*. The sensitivities to lenvatinib were assessed in a panel of HCC cells, including HCCLM3, Huh7, Bel‐7402, SMMC‐7721 and HepG2. A total of 3000 cells were plated in 96‐well plate in triplicate and treated with gradually increasing concentrations (0.00001, 0.0001, 0.001, 0.01, 1, 10 and 100 µm) of lenvatinib (FGFR/VEGFR inhibitor; S1164, Selleck, USA) or/and a constant concentration (2.5 µM) of gefitinib (EGFR inhibitor; S1025, Selleck, USA) for 72 h. Lenvatinib and gefitinib were dissolved in dimethyl sulfoxide (DMSO) for cell viability assays. Cell viability was determined using the CCK‐8 assays and normalized to the DMSO control group and was expressed as a percentage of maximum proliferation.

*In vivo assays in cell line‐derived xenograft (CDX) mouse models*. The 6‐week‐old male nude BALB/c mice (Vital River Laboratories, Beijing, China) were randomly divided into the indicated groups (8 mice/group). To assess the role of *TBC1D31* knockdown on the anti‐tumoral effects of Lenvatinib in TBC1D31^high^ HCCLM3 cells, we constructed the Tet‐On *TBC1D31* shRNA knockdown cell lines. Oligonucleotides targeting *TBC1D31* were synthesized and cloned into pHS‐BSR‐LJ012 vector (Tet‐On inducible knockdown vector). All the constructs used in this study were confirmed by DNA Sanger sequencing. Lentiviruses were produced in HEK293T cells by co‐transfection with the lentiviral‐based construct using the packaging plasmids pSPAX2 and the envelope vector pMD2.G. Forty‐eight hours after transfection, the infectious lentiviruses were harvested and used for the transduction of HCCLM3 cells in the presence of 8 µg/mL polybrene (Sigma, USA). Stable cell pools were selected in medium containing 2 µg/mL puromycin (Merk, USA). The DOX‐inducible Tet‐On‐shTBC1D31‐expressing or Tet‐On‐shCtrl‐expressing HCCLM3 cells were subcutaneously transplanted into one side of mice back (1.0 × 10^6^ cells/mouse). After the tumor volume reached ≈100 mm^3^, 2 mg/mL DOX (Sigma, USA) was administered in drinking water containing 1% sucrose (Sigma, USA) every day. The mice in the two groups were randomized and orally administered with vehicle (0.5% CMC‐Na) or lenvatinib (4 mg k^−1^g) every 5 days. To assess the role of *EGFR* knockdown induced by gefitinib on the anti‐tumoral effects of lenvatinib in TBC1D31^high^ HCCLM3 cells, HCCLM3 cells were subcutaneously transplanted into one side of mice back (1.0 × 10^6^ cells/mouse). After the tumor volume reached ≈100 mm^3^, mice were randomized and orally administered with vehicle (0.5% CMC‐Na), lenvatinib (4 mg k^−1^g), gefitinib (80 mg k^−1^g) or a drug combination in which each compound was administered at the same dose every 5 days. The tumors volumes were measured every three days. When tumors reached a maximum of 1500 mm^3^, mice were euthanized and tumors were harvested and procured for IHC analyses. The tumor growth inhibition rate (TGI%) in drug‐treated mice relative to the controls was calculated as follows: (1 – [tumor volume of treated mice]/[average tumor volume of control mice]) × 100%.

*In vivo assays in patient‐derived tumor xenograft (PDX) mouse models*. A total of 12 HCC patients were recruited from the Fifth Medical Center of Chinese PLA General Hospital (Beijing, China) and the tumor samples were subjected to establishment of PDX mice models by IDMO Co., Ltd. (Beijing, China). Through qPCR analyses of the genomic copy numbers of *TBC1D31* in these samples, two ones (TBC1D31^low^ [without genomic amplification and with low expression level of TBC1D31] and TBC1D31^high^ [with genomic amplification and with high expression level of TBC1D31]) were chosen for further studies. Briefly, Fresh tumor tissues were placed in ice‐chilled high‐glucose DMEM with 10% FBS, 100 U/mL penicillin and 100 U/mL streptomycin and rapidly processed for engraftment. After the removal of necrotic tissue, the tumor specimens were partitioned into 2 × 1 × 1 mm^3^ sections and washed 3 times with ice‐cold PBS. The tissue fragments were incubated in DMEM medium supplemented with 50% Matrigel (356234, BD, USA), 10 ng/mL epidermal growth factor (PHG0314, Gibco, USA), 10 ng/mL basic fibroblast growth factor (PHG0264, Gibco, USA), 100 U/mL penicillin and 100 U/mL streptomycin for 30 min. Three pieces of tumor tissues with the incubation mix (Matrigel plus growth factors) were subcutaneously transplanted into the right flanks of 5‐week‐old male BALB/c nude mice (Vital River Laboratories, Beijing, China). Once the subcutaneous tumor reached 1 cm in diameter, it was minced into pieces (≈2 mm^3^) and then subcutaneously implanted into the flanks of 5‐week‐old male BALB/c nude mice. When the tumor volume reached ≈150 mm^3^ after implantation, mice were randomized (5 mice/group) and orally administered with vehicle (0.5% CMC‐Na), lenvatinib (4 mg k^−1^g), gefitinib (80 mg k^−1^g) or a drug combination in which each compound was administered at the same dose every 5 days. When tumors reached a maximum of 1500 mm^3^, mice were euthanized and tumors were harvested and procured for H&E staining and IHC staining of TBC1D31, EGFR, Ki67 and cleaved Caspase3.

*In vitro assays in patient‐derived primary tumor cell (PDC) models*. To establish the primary HCC cell cultures, the HCC tissues from the above TBC1D31^high^ and TBC1D31^low^ PDX models were minced using a razor blade and digested in collagenase digestion buffer at 37 °C for 1 h. Cells were passed through 100 µm and 40 µm cell strainers and centrifuged at 1,200 rpm for 5 min. Cells were incubated in RBC lysis buffer for 2 min and then re‐suspended in 6 mL medium and spun through 0.5 mL of serum layered on the bottom of the tube to remove the cellular debris. The contaminated human or mouse haematopoietic and endothelial cells were depleted using the biotin‐conjugated anti‐mouse CD45, CD31 and Ter119 antibodies and separated on a MACS LS column using anti‐biotin microbeads. The primary cells were cultured in hepatoma carcinoma cell medium (PreceDo Pharmaceuticals Co., Ltd., Hefei City, China) for validation of drug sensitivity as described in the “In vitro *assays in HCC cell lines*” section.

### Statistical Analyses

All functional assays were performed at least three times. The accompanying quantification and statistics were derived from at least 3 independent replicates, and reported as mean ± standard deviation (s.d.). The differences were assessed using two‐sided Student's *t* test for two‐group comparisons, and ANOVA for multi‐group comparisons. The overall survival (OS) period was defined as the time between the surgery and the death or the last follow‐up examination. The DFS period was calculated from the date of tumor resection until detection of the first HCC recurrence, death, or the last follow‐up examination. Patients who were lost to be followed‐up or died from causes unrelated to HCC were considered as censored events. Survival curves were analyzed by the Kaplan‐Meier method, and *P* values were determined by the log‐rank test. Independent factors for OS or DFS were evaluated by multivariate Cox proportional hazards regression analysis. In all statistical tests, *P* < 0.05 was considered to be statistically significant unless stated otherwise. Statistical analyses were performed using R (3.5.0) software.

## Conflict of Interest

The authors declare no conflict of interest.

## Author Contributions

P.C., H.C., Y.Z., and Q.Z. contributed equally to this work. G.Q.Z. was the overall study principal investigator who conceived the study and obtained the financial supports. G.Q.Z., F.C.H., and P.B.C. designed the study. P.B.C. analyzed the data. H.X.C., Y.Z., and Q.Z. performed most experiments with the help from M.T.S., L.J., B.Q.G., C.M.G., X.Y.L., A.Q.Y. and C.N.Y. Y.Z. and X.W.W. performed the mouse studies. H.X.C., H.H.H., X.Z., R.J.H., L.J., and Y.H.W. performed the drug response assays. Y.F.L., A.F.S., Q.F.S., H.L., and H.Z.J. recruited the HCC samples and collected the clinical information. P.B.C., H.X.C., and Y.Z. conducted the sample selection and data management, performed the statistical analyses, interpreted the results, and drafted and synthesized the manuscript. G.Q.Z. approved the final version of the manuscript.

## Supporting information

Supporting Information

Supporting Information Tables

## Data Availability

The data that support the findings of this study are available on request from the corresponding author. The data are not publicly available due to privacy or ethical restrictions.
