# Supplementary material for: Genomic Amplification of TBC1D31 Promotes Hepatocellular Carcinoma Through Reducing the Rab22A‐Mediated Endolysosomal Trafficking and Degradation of EGFR
Source: Adv Sci (Weinh). 2024 Aug 29;11(40):2405459. doi: 10.1002/advs.202405459 (PMC11516053; doi:10.1002/advs.202405459)
Supplement: Supplementary file 1 — Supporting Information [file ADVS-11-2405459-s002.docx]

**Supporting Information**

**Genomic amplification of *TBC1D31* promotes** **hepatocellular carcinoma through** **reducing the** **Rab22A-****mediated endolysosomal trafficking and degradation of EGFR**

**Running Title:** TBC1D31 promotes HCC by activating the EGFR pathway

Pengbo Cao^1,†^, Hongxia Chen^1,†^, Ying Zhang^2,†^, Qi Zhang^1,3,†^, Mengting Shi^4^, Huihui Han^1^, Xiaowen Wang^5^, Liang Jin^1^, Bingqian Guo^1^, Rongjiao Hao^6^, Xi Zhao^1^, Yuanfeng Li^1^ , Chengming Gao^1^, Xinyi Liu^1^, Yahui Wang^1^, Aiqing Yang^1^, Chenning Yang^1^, Anfeng Si^7^, Hua Li^8^, Qingfeng Song^9^, Fuchu He^2,5,*^, and Gangqiao Zhou^1,2.3,4,6,*^

^1^State Key Laboratory of Medical Proteomics, National Center for Protein Sciences at Beijing, Beijing Institute of Radiation Medicine, Beijing, 100850, China;

^2^School of Life Sciences, Tsinghua University, Beijing, 100084, China;

^3^University of South China, Hengyang City, 421001, China;

^4^Guangxi Medical University, Nanning City, 530021, China;

^5^State Key Laboratory of Medical Proteomics, National Center for Protein Sciences at Beijing, Beijing Institute of Lifeomics, Beijing, 102206, China;

^6^Hebei University, Baoding City, 071000, China;

^7^Jinling Hospital, Affiliated Hospital of Medical School, Nanjing University, Nanjing City, 210002, China;

^8^Department of Oncology, Chengdu Military General Hospital, Chengdu City, 610083, China;

^9^Affiliated Cancer Hospital of Guangxi Medical University, Nanning City, 530021, China;

^†^These authors contributed equally to this work.

**^*^Correspondence should be addressed to:**

Dr. Gangqiao Zhou, The State Key Laboratory of Medical Proteomics, National Center for Protein Sciences at Beijing, Beijing Institute of Radiation Medicine, 27 Taiping Road, Beijing, 100850, P. R. China. E-mail: zhougq114@126.com; Phone & fax: 86-10-66931201.

OR

Dr. Fuchu He, The State Key Laboratory of Medical Proteomics, National Center for Protein Sciences at Beijing, Beijing Institute of Lifeomics, 38 Life Science Road, Beijing, 102206, P. R. China. E-mail: hefc@nic.bmi.ac.cn; Phone & fax: 86-10-68177417.**This file includes:**

**Supplemental Figures 1 - 9:**

Figure S1 The landscape of CNAs in the HCC genomes and the 8q24.13 amplification associated with the prognoses of multiple cancers.

Figure S2 High-content screening of genes within the 8q24.13 amplification and the dysregulation of TBC1D31 in HCC.

Figure S3 High expression of *TBC1D31* predicts poor clinical outcomes in patients with various types of cancer.

Figure S4 TBC1D31 overexpression promotes the growth and metastasis of HCC.

Figure S5 TBC1D31 activates the EGFR pathway in HCC.

Figure S6 TBC1D31 reduces the cell membrane-bounded EGFR.

Figure S7 TBC1D31 exerts its oncogenic role dependent on Rab22A.

Figure S8 TBC1D31 promotes the malignant phenotypes and EGFR pathway activation of HCC cells dependent on Rab22A.

Figure S9 Downregulating TBC1D31 sensitizes the HCC cells to lenvatinib treatment.

**Supplemental Tables 1 - 10:**

Table S1 Summary of the transcriptomic and genomic datasets from the six discovery cohorts.

Table S2 Significant focal CNAs identiﬁed by ACE analyses in the discovery cohorts.

Table S3 Survival analyses of the 15 significant focal CNAs in three discovery cohorts.

Table S4 Major demographic and clinical characteristics of HCC patients in the validation (VALI) cohort.

Table S5 Characteristics of HCC patients in the validation (VALI) cohort grouped by 8q24.13 copy numbers or TBC1D31 expression levels.

Table S6 Cox hazard ratios for survival rates of HCC patients grouped by 8q24.13 copy number from the VALI cohort.

Table S7 Cox hazard ratios for survival rates in HCC patients grouped by TBC1D31 expression from the VALI cohort.

Table S8 GSEA based on the TBC1D31 expression levels in HCC tissues from the discovery cohorts.

Table S9 GSEA based on the dependency score correlation between TBC1D31 and the other genes.

Table S10 Primers, shRNAs and siRNAs in this study.**Supplemental Figures**


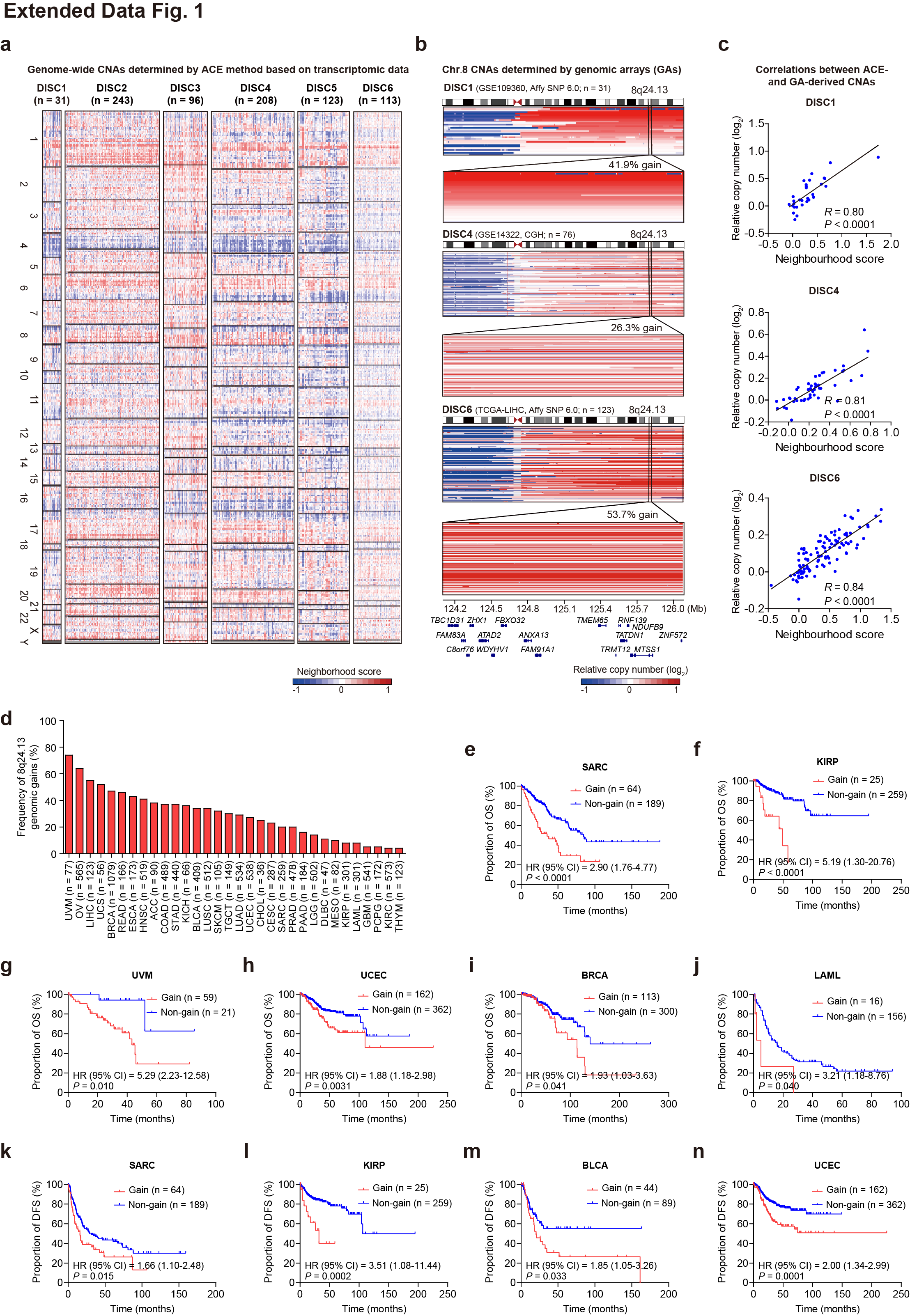


**Figure S1 The landscape of CNAs in the HCC genomes and the 8q24.13 amplification associated with the prognoses of multiple cancers.**

**a**) The heatmap of the whole-genome neighborhood scores (NSs), which were generated by the analysis of CNAs by expression data (ACE) method in the six discovery cohorts. CNA, copy number alteration. **b**) Genomic amplification at chromosome 8q24.13 (in red) in HCCs based on the genomic arrays data sets from the DISC1 (GSE109360, by Affymetrix SNP 6.0 array), DISC4 (GSE14332, by comparative genomic hybridization [CGH] arrays) and DISC6 (from the Cancer Genome Atlas [TCGA], generated by single nucleotide polymorphism [SNP] arrays) cohorts, respectively. Color bar represents the scale of copy number gain (red) and loss (blue) on the basis of log ratio (LR) segment mean. **c**) The correlation between the ACE-inferred NSs and the genomic array (GA)-generated relative copy numbers (LR segment mean) at 8q24.13 in the DISC1, DISC4 and DISC6 cohorts. The *R* and *P* values were determined by Pearson correlation analyses. **d**) The frequencies of the 8q24.13 genomic gain (LR segment mean > 0.3) across multiple types of cancer, which were determined by the genomic data (generated by SNP arrays) from TCGA pan-cancer cohorts. The abbreviations for those tumor names can be found on the TCGA project website. **e**-**n**) Kaplan-Meier analyses showing that the genomic gain of 8q24.13 in tumor tissues is significantly correlated with lower overall survival (OS) or disease-free survival (DFS) rates in several types of human cancer from the TCGA project. The hazard ratios (HR) were calculated using univariate Cox proportional hazards regression analyses. *P* values were determined by log-rank tests.


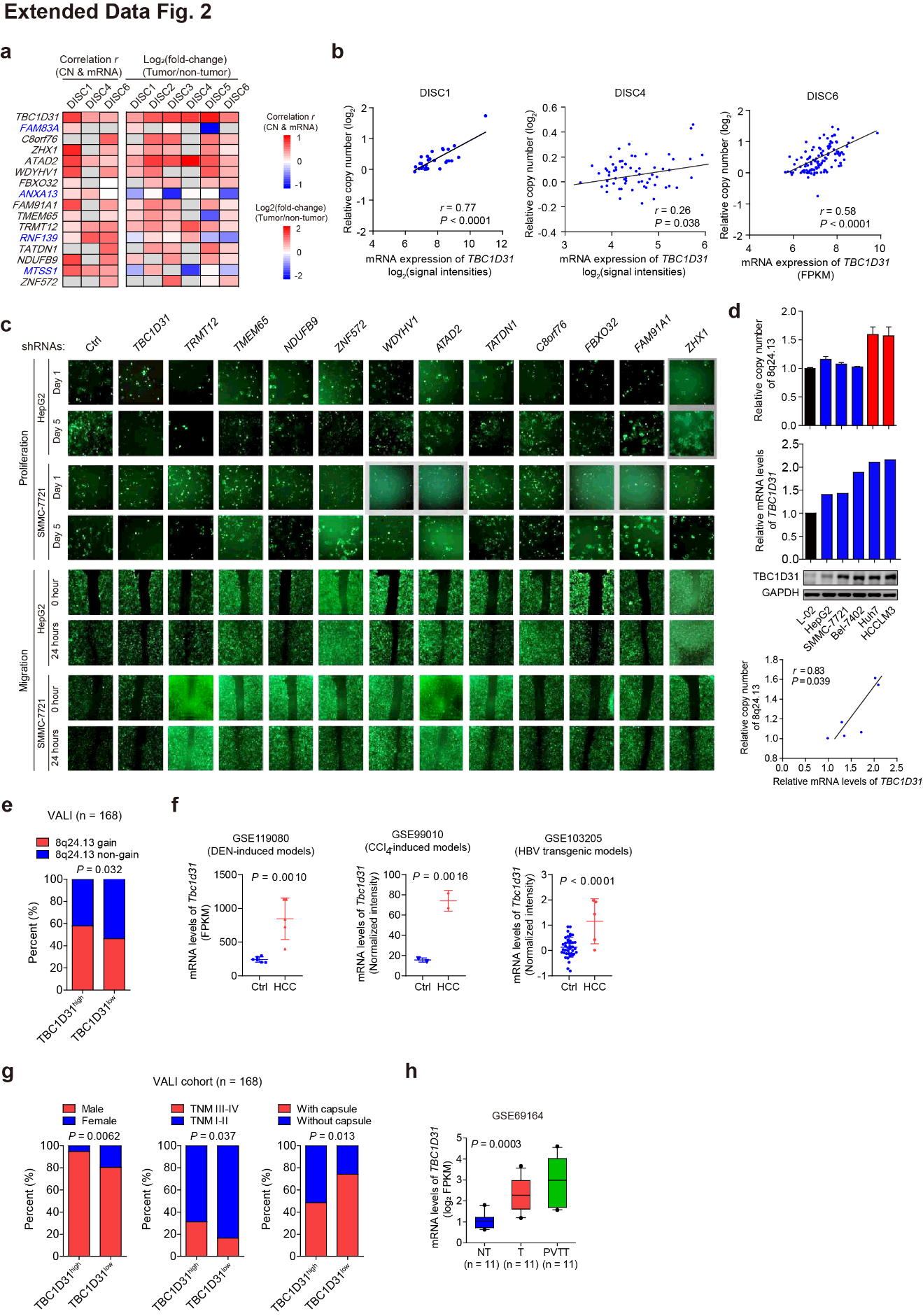


**Figure S2 High-content screening of genes within the 8q24.13 amplification and the dysregulation of TBC1D31 in HCC.**

**a**) Correlations between the mRNA levels of genes within 8q24.13 amplification and 8q24.13 copy numbers (CNs), and the differential expression of these genes between the HCC tissues and matched non-tumor liver tissues. The CN-mRNA correlations were assessed by Pearson correlation analysis. The relative fold-changes of mRNA expression levels of these genes between HCC tissues and non-tumor liver tissues were examined. **b**) Correlations between the mRNA levels of *TBC1D31* and the copy numbers of 8q24.13 determined by genotyping arrays or CGH arrays (log ratio [LR] segment mean) in HCC tumors from the DISC1, 4 and 6 cohorts, by Spearman correlation analyses. **c**) The representative results of high-content screening (HCS) assays for those 12 candidate genes within 8q24.13 amplification in HepG2 and SMMC-7721 cells. Up panel, cell number counting assays were used for assessing the ability of cell proliferation on the basis of the images taken at the 1st and 5th days post transfection. Down panel, wound-healing assays were used for assessing the ability of cell migration. Cell monolayers were scratched and the images were taken at 0 and the 24th hours after wounding. All the experiments were performed in triplicates after transfection with the indicated siRNA pools targeting each individual gene in HepG2 and SMMC-7721 cells, respectively. Ctrl, control. **d**) Correlation between the 8q24.13 copy numbers and *TBC1D31* mRNA levels in a panel of liver cell lines, including one normal immortalized human fetal hepatocyte (L-02) and five types of HCC cell line (HepG2, SMMC-7721, Bel-7402, Huh7 and HCCLM3). The relative mRNA expression levels of *TBC1D31* were assessed by quantitative reverse transcription PCR (qRT-PCR) assays and normalized to reference *GAPDH*. The relative copy number of 8q24.13 amplification was determined by quantitative PCR (qPCR) assays and normalized by the average genomic content of *LTBP1* (at 2p22.2 locus), *SATB1* (3p24.3) and *ANO3* (11p14.3). **e**) Correlation between the TBC1D31 protein expression levels and 8q24.13 copy number gain of HCC patients from the VALI cohort. Samples with the IHC score of TBC1D31 greater than 5 were defined as the TBC1D31^high^ group, while the others as the TBC1D31^low^ group. *P* values were determined by chi-square test. **f**) *Tbc1d31* mRNA levels in three datasets of murine HCC models. Left, the DEN-induced mouse models, which include 6 non-tumor livers (Ctrl) and 5 tumors (HCC) dissected from mice (GSE119080 dataset); Middle, the Western diet CCl4-induced mouse models, which include 2 non-tumor livers and 2 tumors dissected from mice at the 24th weeks (GSE99010); Right, the hepatitis B virus (HBV) transgenic mouse models, which include a collection of non-tumor liver tissues (n = 40) at the 7th days, and 1st, 3rd, 6th, 9th, 12th, 15th and 18th months, and 5 tumors dissected from mice at the 18th months (GSE103205). **g**) Correlations between the TBC1D31 protein levels and gender (left), tumor-node-metastasis (TNM) stage (middle) or tumor capsule status (right) of HCC patients from the VALI cohort. *P* values were determined by chi-square test. **h**) *TBC1D31* mRNA levels in the matched portal vein tumor thrombus (PVTT), non-tumor liver tissues (NT) and primary tumor tissues (T), which were derived from a RNA sequencing dataset from a previous study (GSE69164; n = 11/each tissue type). *P* values were determined by analysis of variance (ANOVA) test. FPKM, fragments per kilobase of transcript per million mapped reads.


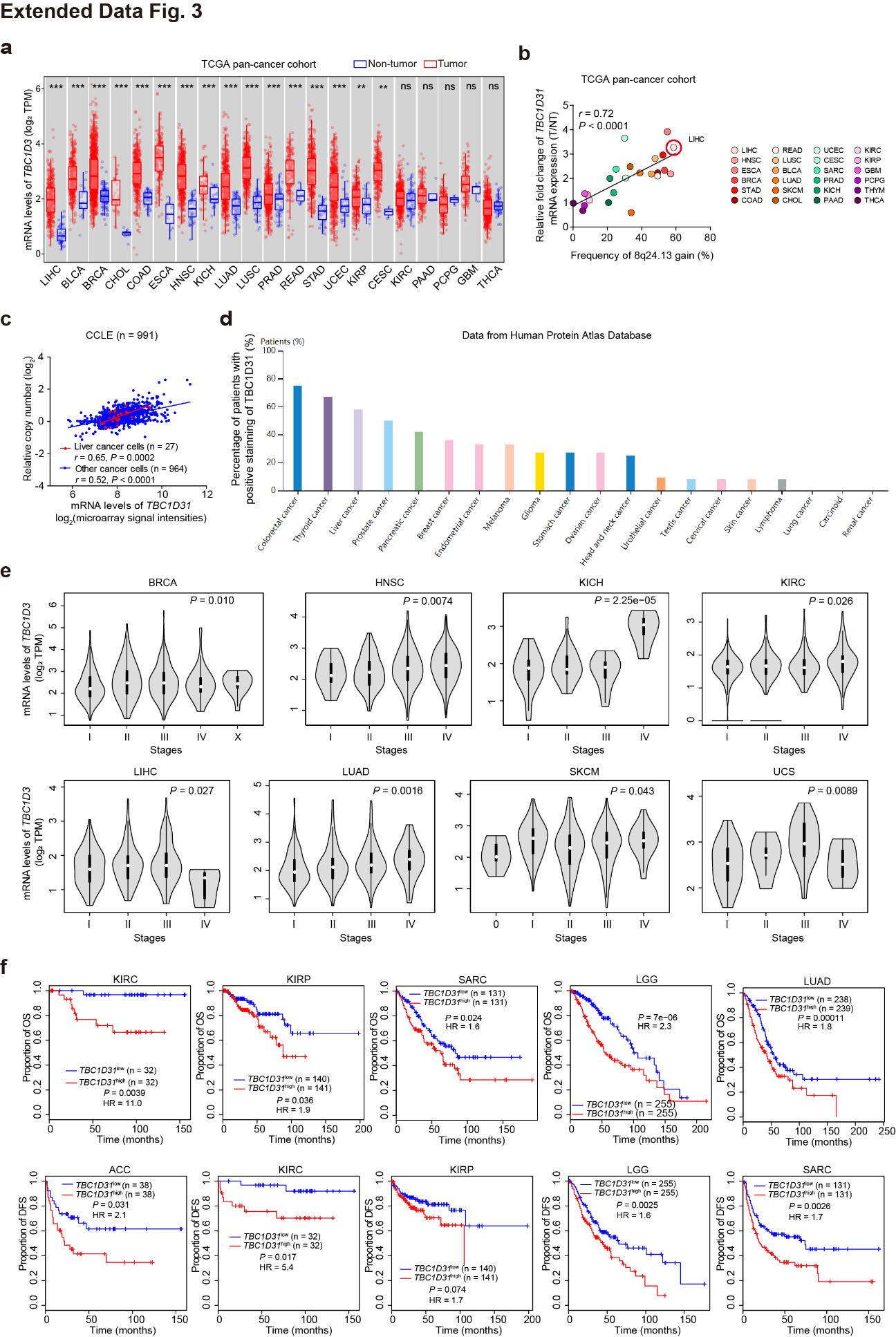


**Figure S3 High expression of *TBC1D31* predicts poor clinical outcomes in patients with various types of cancer.**

**a**) The *TBC1D31* mRNA levels in tumor and non-tumor tissues from the TCGA pan-cancer cohorts. In most types of cancer, the expression of TBC1D31 is significantly higher in tumor tissues than in non-tumor tissues. The abbreviations for those tumor names can be found on the TCGA project website. TPM, transcript per million; TCGA, the Cancer Genome Atlas. *P* values were calculated using Student’s *t* test unless specifically specified. ^***^, *P* < 0.001; ^**^, *P* < 0.01; n.s., not significant. **b**) The prevalence of 8q24.13 gain (LR segment mean > 0.3) was positively correlated with the average fold-change of *TBC1D31* mRNA levels (tumor/non-tumor) in cancers from the TCGA pan-cancer cohorts. **c**) The copy numbers of 8q24.13 (LR segment mean) were positively correlated with *TBC1D31* mRNA levels in a collection of HCC (n = 27) and other types of cancer cell lines (n = 964) from the Cancer Cell Line Encyclopedia (CCLE) project. **d**) TBC1D31 protein expression in multiple cancer types from the Human Protein Atlas database. The percentage of patients with positive staining of TBC1D31 was determined by immunohistochemistry assays. **e**) *TBC1D31* mRNA levels are correlated with the advanced clinical stages in several types of cancer from TCGA project*. P* values were determined by analysis of variance (ANOVA) test. **f**) Kaplan-Meier analyses showing that higher TBC1D31 expression levels in tumor tissues are significantly correlated with lower overall survival (OS) or disease-free survival (DFS) rates in several types of cancer from the TCGA project. The patients with *TBC1D31* mRNA expression levels greater than the median value in tumor tissues were defined as the *TBC1D31*^high^ group, while the others were *TBC1D31*^low^ group. The hazard ratios (HR) were calculated using the univariate Cox proportional hazards regression analyses. *P* values were determined by log-rank tests.
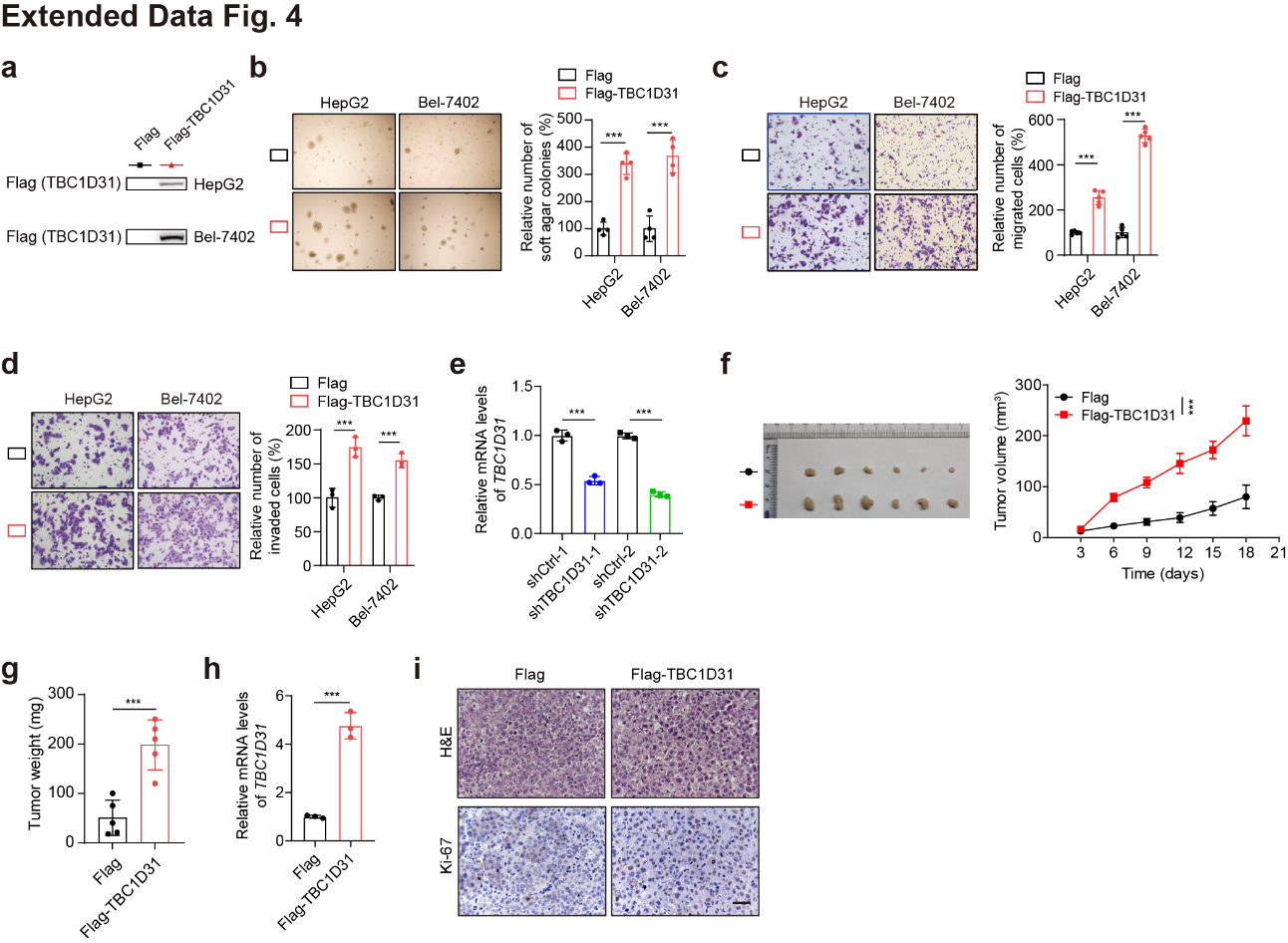


**Figure S4 TBC1D31 overexpression promotes the growth and metastasis of HCC.**

**a**) Immunoblotting assays showing the efficiencies of TBC1D31 overexpression in HepG2 and Bel-7402 cells. **b**-**d**) The effects of TBC1D31 overexpression on soft agar cell colony formation (**b**), migration (**c**) and invasion (**d**) in HepG2 and Bel-7402 cells. **e**) The efficiencies of *TBC1D31* knockdown (by sh*TBC1D31*-1 or sh*TBC1D31*-2) in HCCLM3-derived subcutaneous tumor tissues from the nude mice, which were determined by quantitative reverse transcription PCR (qRT-PCR) assays. **f**-**i**) The effects of TBC1D31 overexpression on tumors growth in nude mice. Control empty vector-expressing or TBC1D31-expressing HepG2 cells were implanted subcutaneously at the back of the mice (n = 6 mice/group). The representative images of subcutaneous tumors and dynamic change of tumor volume (**f**), tumor weights (**g**), efficiencies of TBC1D31 overexpression in subcutaneous tumor tissues (**h**), and the hematoxylin and eosin (H&E) staining and immunohistochemistry (IHC) staining of Ki-67 in subcutaneous tumors (**i**) were shown, respectively. Scale bars, 200 μm.

Values are expressed as mean ± standard deviation (s.d.) of three or more independent replicates. *P* values were calculated using Student’s *t* test unless specifically specified. ^***^, *P* < 0.001; n.s., not significant.


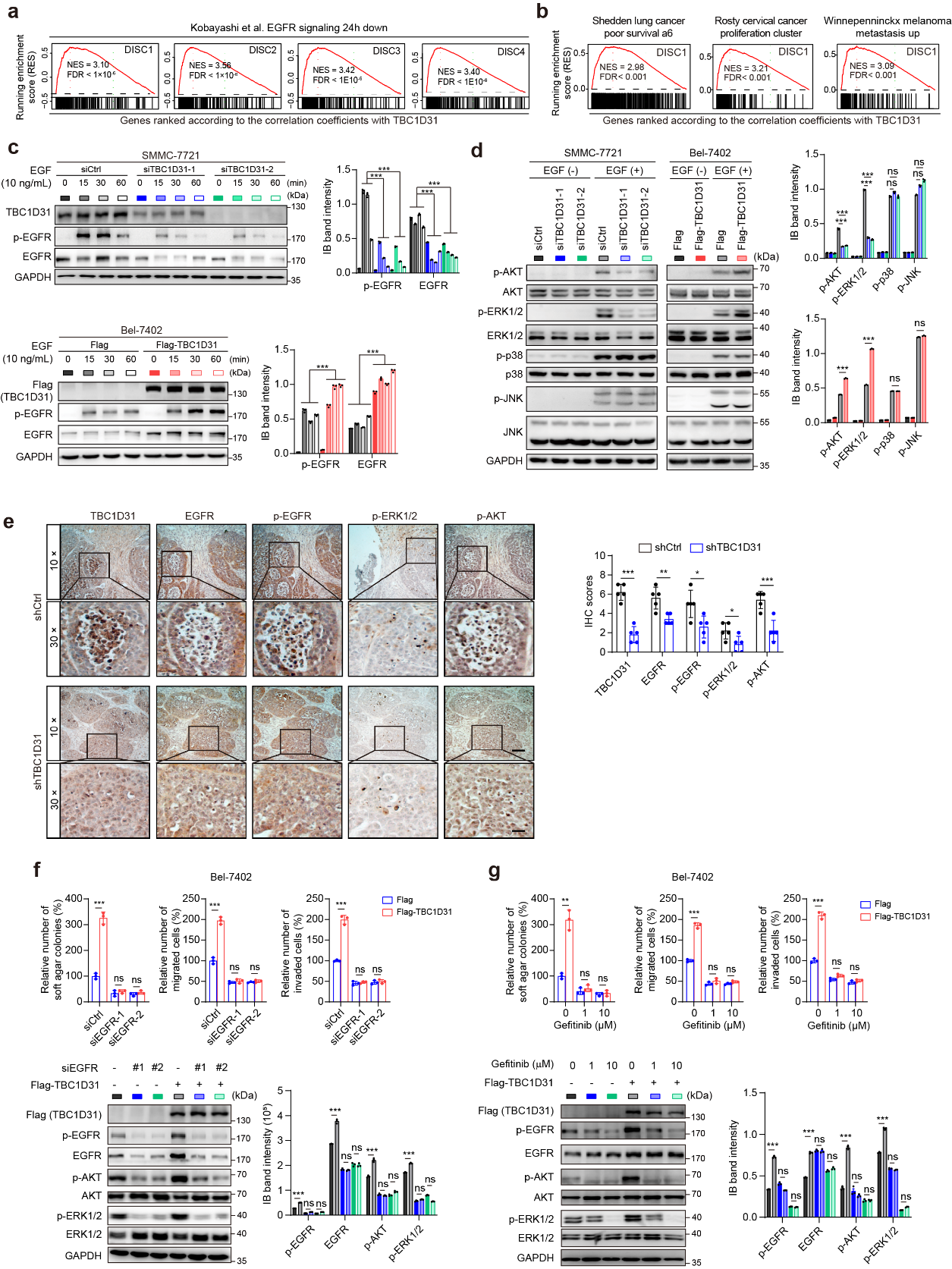


**Figure S5 TBC1D31 activates the EGFR pathway in HCC.**

**a**) GSEA plots of running enrichment score of the “Kobayashi et al. EGFR signaling 24h down” in the four discovery HCC cohorts (DISC1, 2, 3 and 4). For each dataset, the Pearson correlation coefficients between the expression levels of *TBC1D31* and each of the other genes were computed and then the genes were ranked according to the coefficients. The rank list was used to identify the significantly enriched gene sets by using GSEA. The significance was assessed by 1,000 permutations with gene labels sampling. FDR, false discovery rate; GSEA, gene set enrichment analysis; NES, normalized enrichment score. **b**) GSEA plots of running enrichment score of the proliferation- and metastasis-related gene sets based on the transcriptomic dataset from the DISC1 cohort. **c**) The effects of knockdown (by si*TBC1D31*-1 or si*TBC1D31*-2) or overexpression of TBC1D31 on the protein levels of EGFR and phosphorylated-EGFR (p-EGFR, Tyr1068) in SMMC-7721 and Bel-7402 cells, respectively. The cells were treated with EGF (10 ng/mL) for 0, 15, 30 and 60 minutes (min) after serum starvation. **d**) The effects of *TBC1D31* knockdown or overexpression on AKT/p-AKT (Ser473), ERK1/2/p-ERK1/2 (Thr202/Tyr204), p38/p-p38 (Thr180/Tyr182), JNK/p-JNK (Thr183/Tyr185) protein levels in SMMC-7721 and Bel-7402 cells, respectively, without or with EGF treatment (10 ng/mL) for 30 min. **e**) The effects of *TBC1D31* knockdown in HCCLM3 cells on the levels of EGFR, p-EGFR, p-ERK1/2 and p-AKT in subcutaneous tumor tissues from the nude mice by immunohistochemistry (IHC) analyses. Scale bars, 100 μm. **f**) Knockdown of *EGFR* by siRNAs targeting *EGFR* (siEGFR-1 or siEGFR-2) abolishes the promoting effects of TBC1D31 overexpression on abilities of cells soft agar colony formation, migration and invasion, and the levels of p-AKT and p-ERK1/2 in HepG2 and Bel-7402 cells. The cells were treated with EGF (10 ng/mL) for 30 min after serum starvation. **g**) Inhibition of the EGFR pathway activity by EGFR inhibitor gefitinib (0, 1 and 10 µM) abolishes the promoting effects of TBC1D31 overexpression on the abilities of cells soft agar colony formation, migration and invasion, and the levels of p-AKT and p-ERK1/2 in HepG2 and Bel-7402 cells. The cells were treated with EGF (10 ng/mL) for 30 min after serum starvation.

Values are expressed as mean ± standard deviation (s.d.) of three or more independent replicates. *P* values were calculated using Student’s *t* test unless specifically specified. ^***^, *P* < 0.001; n.s., not significant.


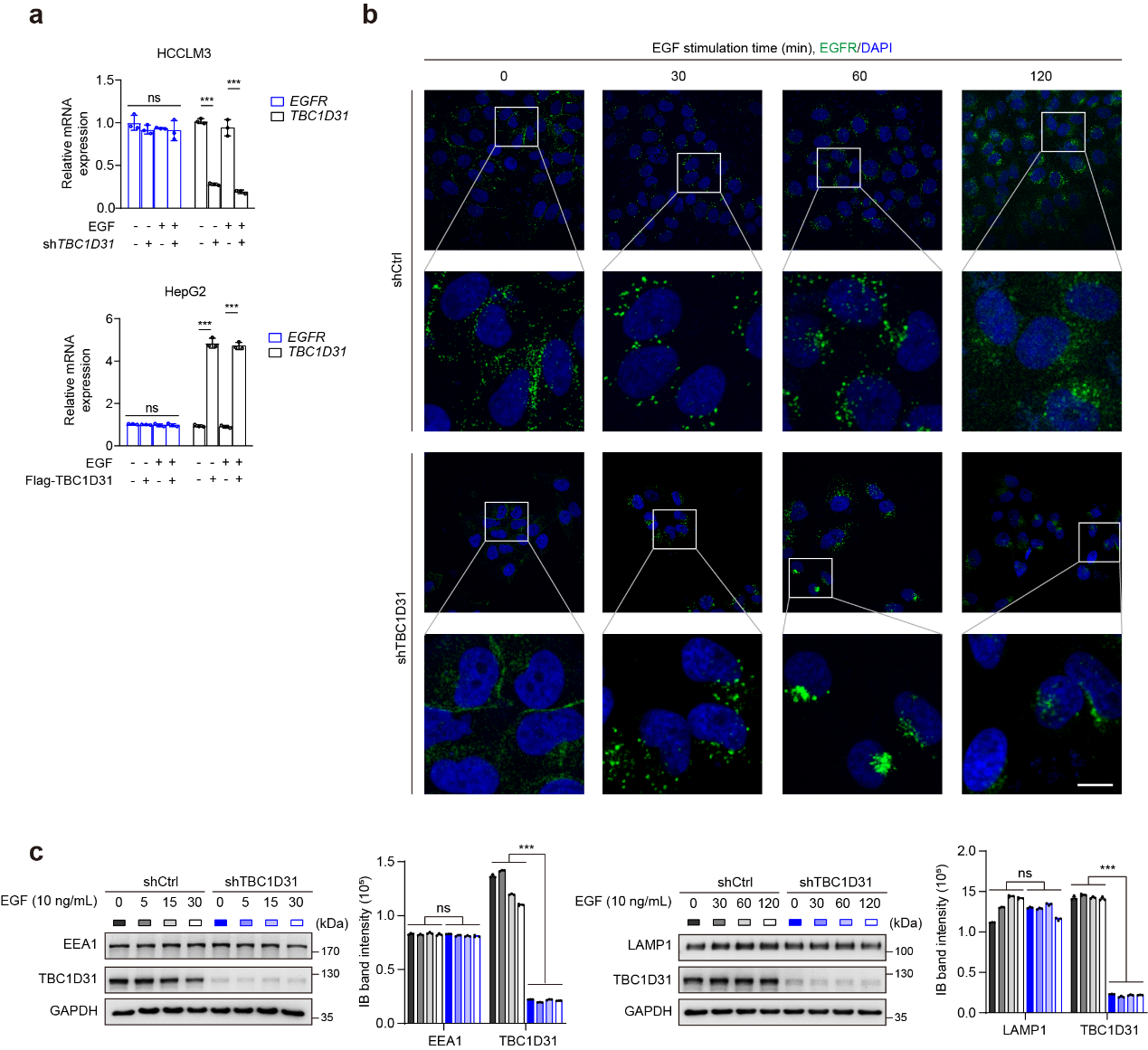


**Figure S6 TBC1D31 reduces the cell membrane-bounded EGFR.**

**a**) Either knockdown (left) or overexpression (right) of TBC1D31 does not affect *EGFR* mRNA levels in HCCLM3 and HepG2 cells, regardless of whether EGF treatment (10 ng/mL) is used. **b**) Knockdown of *TBC1D31* reduced the cell membrane-bounded EGFR in HCCLM3 cells by immunofluorescence assays. The HCCLM3 cells were serum-starved and stimulated with EGF (10 ng/mL) for the indicated times. Immunofluorescence assays were performed using primary anti-EGFR antibody followed with Alexa Fluor-488 conjugated secondary antibody. **c)** Knockdown of TBC1D31 doesn’t affect the protein expression levels of EEA1 and LAMP1 in HCCLM3 cells. The HCCLM3 cells were serum-starved and stimulated with EGF (10 ng/mL) for the indicated times.

Values are expressed as mean ± standard deviation (s.d.) of three or more independent replicates. *P* values were calculated using Student’s *t* test unless specifically specified. ^***^, *P* < 0.001; n.s., not significant.


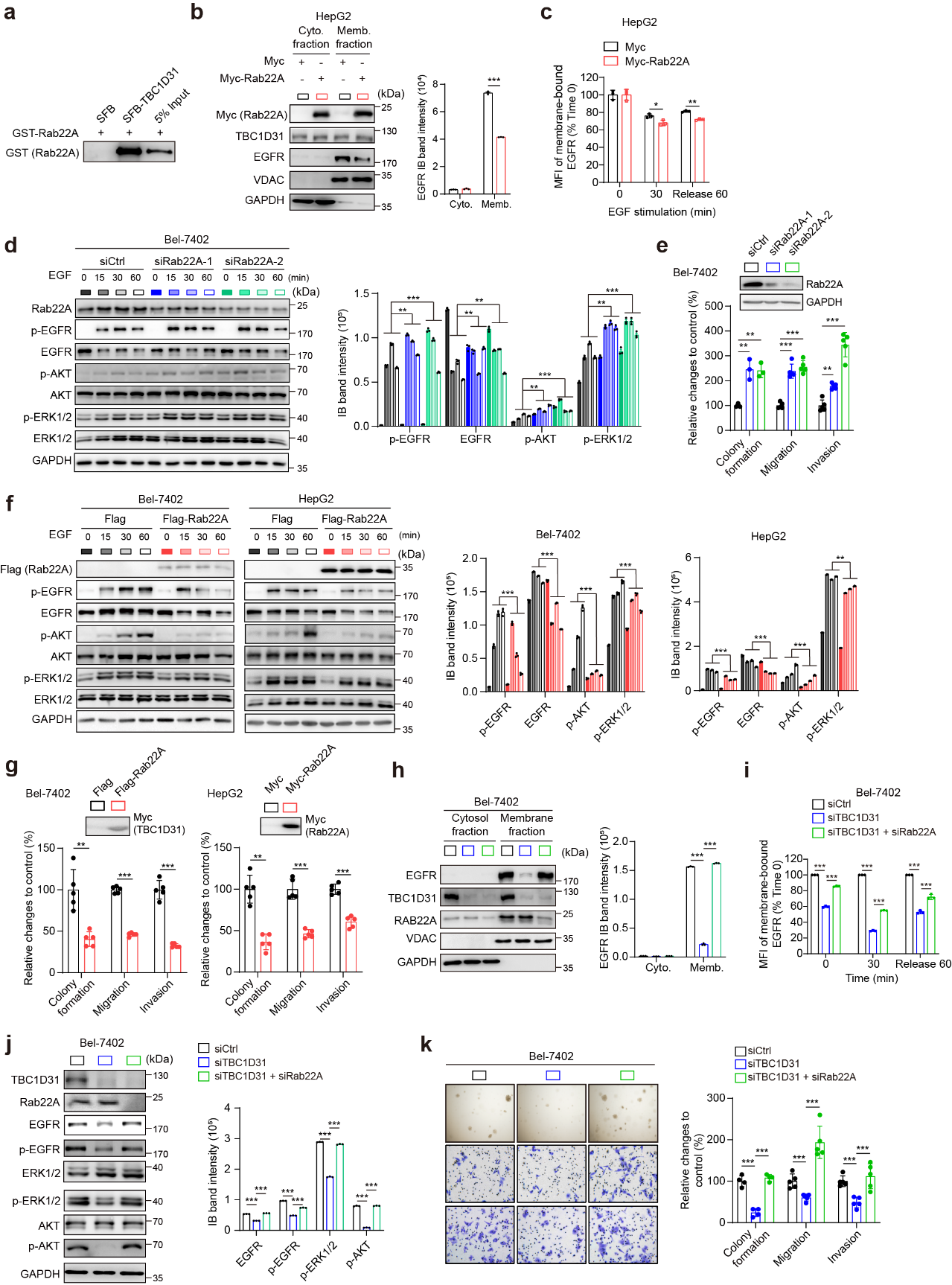


**Figure S7 TBC1D31 exerts its oncogenic role dependent on Rab22A.**

**a**) The *in vitro* interaction between TBC1D31 (SFB-tagged) and Rab22A (GST-tagged) was evaluated by SFB pull-down followed by immunoblotting assays using an antibody against GST. **b**,**c**) The effect of *Rab22A* knockdown on the role of *TBC1D31* knockdown in reducing the levels of EGFR on the cell membrane, as assessed by cell fractionation assays (**b**) and flow cytometry assays (**c**) in Bel-7402 cells. **d**) The effect of *Rab22A* knockdown on the levels of total and phosphorylated EGFR (p-EGFR), and the activation of downstream cascades of the EGFR pathway in Bel-7402 cells. Protein levels were determined at 30 minutes (min) after EGF stimulation (10 ng/mL). **e**) The effect of *Rab22A* knockdown on the abilities of cells soft agar colony formation, migration and invasion in HCCLM3 and Bel-7402 cells. **f**) The effect of Rab22A overexpression on the levels of total and phosphorylated EGFR (p-EGFR), and the activation of downstream cascades of the EGFR pathway in Bel-7402 and HepG2 cells. Protein levels were determined at 30 minutes (min) after EGF stimulation (10 ng/mL). **g**) The effect of Rab22A overexpression on the abilities of cells soft agar colony formation, migration and invasion in Bel-7402 and HepG2 cells. **h**,**i**) The effect of *Rab22A* knockdown on the role of *TBC1D31* knockdown in reducing the levels of EGFR on the cell membrane, as assessed by cell fractionation assays (**h**) and flow cytometry assays (**i**) in Bel-7402 cells. **j**) The effect of *Rab22A* knockdown on the role of *TBC1D31* knockdown in attenuating the levels of total and phosphorylated EGFR (p-EGFR), and the activation of downstream cascades of the EGFR pathway in Bel-7402 cells. Protein levels were determined at 30 minutes (min) after EGF stimulation (10 ng/mL). **k**) The effect of *Rab22A* knockdown on the role of *TBC1D31* knockdown in attenuating the abilities of cells soft agar colony formation, migration and invasion in HCCLM3 and Bel-7402 cells.

Values are expressed as mean ± standard deviation (s.d.) of three or more independent replicates. *P* values were calculated using Student’s *t* test unless specifically specified. ^*^, *P* < 0.05; ^**^, *P* < 0.01; ^***^, *P* < 0.001.


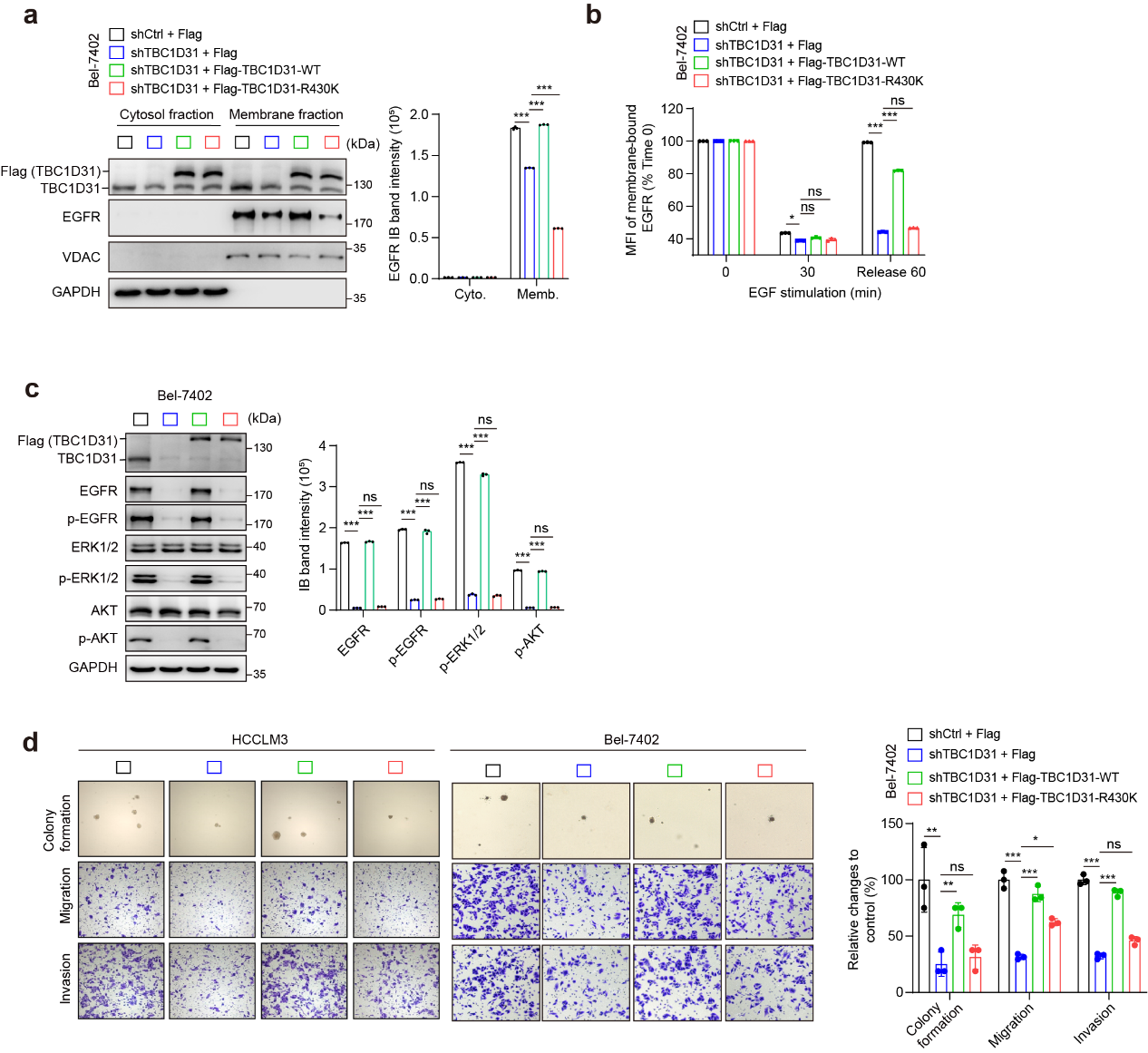


**Figure S8 TBC1D31 promotes the malignant phenotypes and EGFR pathway activation of HCC cells dependent on Rab22A.**

**a**,**b**) Rescue assays showing the effects of re-introduction of WT or R430K mutant TBC1D31 on the levels of EGFR on the cell membrane, as assessed by cell fractionation assays (**a**) and flow cytometry assays (**b**) in *TBC1D31*-knocked-down Bel-7402 cells. **c**) Rescue assays showing the effects of re-introduction of WT or R430K mutant TBC1D31 on the levels of total EGFR and p-EGFR, and the activation of downstream cascades of the EGFR pathway in *TBC1D31*-knocked-down Bel-7402 cells. **d**) Rescue assays showing the effects of re-introduction of WT or R430K mutant TBC1D31 on the abilities of cells soft agar colony formation, migration and invasion in *TBC1D31*-knocked-down HCCLM3 and Bel-7402 cells.

Values are expressed as mean ± standard deviation (s.d.) of three or more independent replicates. *P* values were calculated using Student’s *t* test unless specifically specified. ^*^, *P* < 0.05; ^**^, *P* < 0.01; ^***^, *P* < 0.001; n.s., not significant.
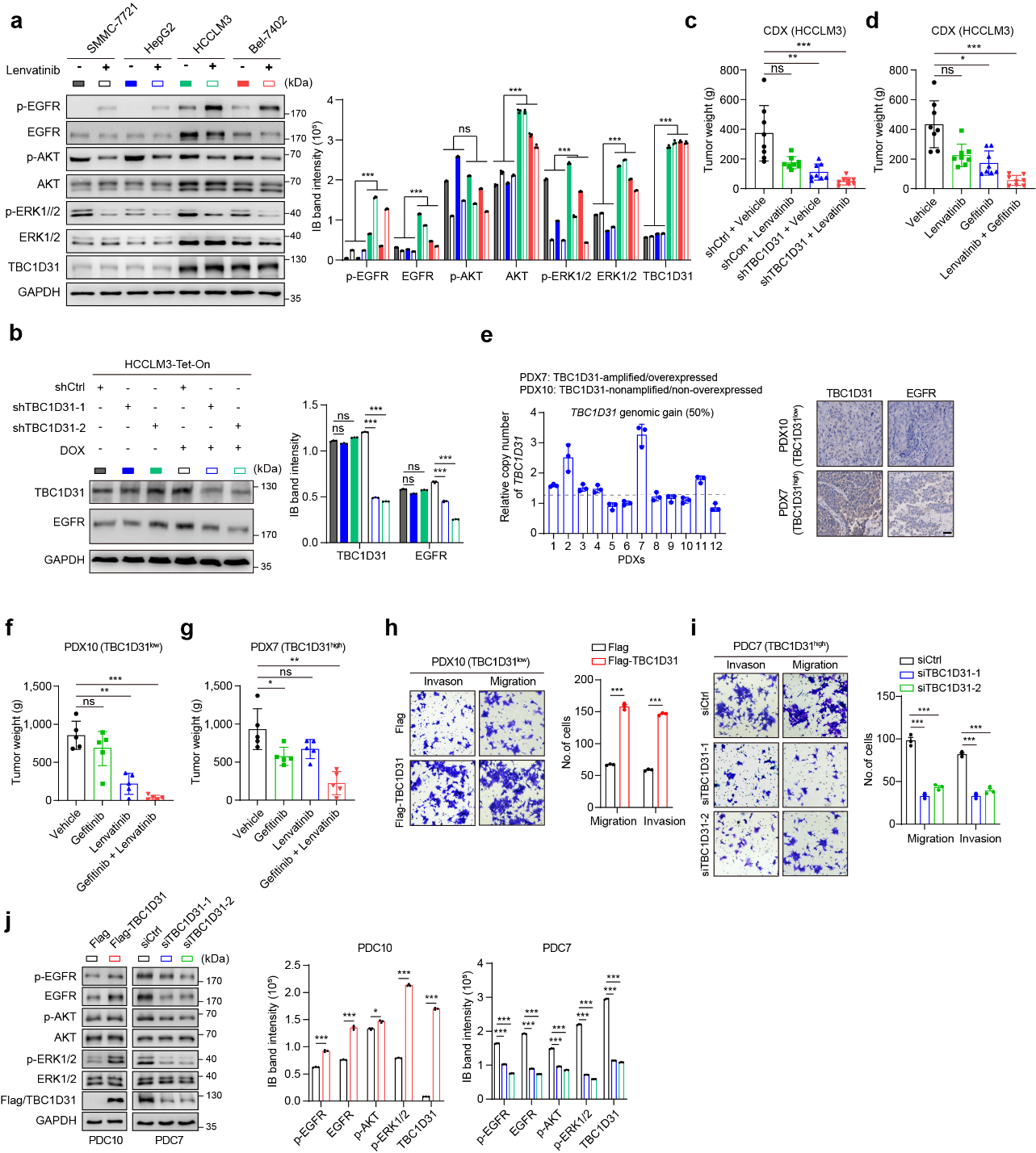


**Figure S9 Downregulating TBC1D31 sensitizes the HCC cells to lenvatinib treatment.**

**a**) The effects of lenvatinib (5 µM, for 4 hours) treatment on the activation of the EGFR pathway in HepG2 and SMCC-7721 cells (with lower TBC1D31 expression, designated as TBC1D31^low^), or HCCLM3 and Bel-7402 cells (with higher TBC1D31 expression, designated as TBC1D31^high^ ). **b**) The effect of Tet-On sh*TBC1D31* system on the knockdown of *TBC1D31* in HCCLM3 cells with or without doxycycline (DOX) treatment (100 ng/mL). **c**) The effects of lenvatinib (4 mg/kg) or *TBC1D31* knockdown or their combination on tumor weights of the dissected tumors in **Fiure 7f**. **d**) The effects of lenvatinib (4 mg/kg) or gefitinib (80 mg/kg) or their combination on tumor weights of the dissected tumors in **Figure** **7g**. **e**) The relative genomic copy numbers of *TBC1D31* in tumor tissues from a total of 12 HCC PDX models. The tumors from the patient 7 with genomic amplification and high levels of TBC1D31 (TBC1D31^high^) and EGFR, and patient 10 with diploid and low levels of TBC1D31 (TBC1D31^low^) and EGFR were selected for drug sensitivity tests. PDX, patient-derived xenograft. The protein levels of TBC1D31 and EGFR in the PDX7 and PDX10 models were determined by IHC assays. The relative copy number of *TBC1D31* was determined by qPCR assays and a relative copy number > 1.25 was defined as genomic gain. Scale bar, 200 μm. **f**) The effects of lenvatinib (4 mg/kg) or gefitinib (80 mg/kg) or their combination on the weights of the dissected tumors in **Figure 7h**. **g**) The effects of lenvatinib (4 mg/kg) or gefitinib (80 mg/kg) or their combination on the weights of the dissected tumors in **Figure 7i**. **h**) The effects of TBC1D31 overexpression on the abilities of cells migration and invasion in the TBC1D31^low^ patient-derived primary tumor cells (PDC10). **i**) The effects of *TBC1D31* knockdown in the TBC1D31^high^ patient-derived primary tumor cells (PDC7) on the abilities of cells migration and invasion. **j**) The effects of *TBC1D31* knockdown in the TBC1D31^high^ PDC7 cells and TBC1D31 overexpression in the TBC1D31^low^ PDC10 cells on the levels of EGFR, p-EGFR, p-AKT and p-ERK1/2.

Values are expressed as mean ± standard deviation (s.d.) of three or more independent replicates. *P* values were calculated using Student’s *t* test unless specifically specified. ^*^, *P* < 0.05; ^**^, *P* < 0.01; ^***^, *P* < 0.001; n.s., not significant.

**Supplemental Tables**

All the supplementary tables for this manuscript are provided as Excel files.
